# Recruitment of transcription factor ETS1 to activated accessible regions promotes the transcriptional program of cilia genes

**DOI:** 10.1093/nar/gkad506

**Published:** 2023-06-16

**Authors:** Donghui Zhang, Chong Zhang, Yanmei Zhu, Haixia Xie, Caifeng Yue, Mingfeng Li, Wenlu Wei, Yu Peng, Guibin Yin, Yunmiao Guo, Yiting Guan

**Affiliations:** Zhanjiang Institute of Clinical Medicine, Central People's Hospital of Zhanjiang, Guangdong Medical University Zhanjiang Central Hospital, Zhanjiang 524045, PR China; Zhanjiang Institute of Clinical Medicine, Central People's Hospital of Zhanjiang, Guangdong Medical University Zhanjiang Central Hospital, Zhanjiang 524045, PR China; Zhanjiang Institute of Clinical Medicine, Central People's Hospital of Zhanjiang, Guangdong Medical University Zhanjiang Central Hospital, Zhanjiang 524045, PR China; Precision Clinical Laboratory, Central People's Hospital of Zhanjiang, Guangdong Medical University Zhanjiang Central Hospital, Zhanjiang 524045, PR China; Precision Clinical Laboratory, Central People's Hospital of Zhanjiang, Guangdong Medical University Zhanjiang Central Hospital, Zhanjiang 524045, PR China; Department of Laboratory Medicine, Central People's Hospital of Zhanjiang, Guangdong Medical University Zhanjiang Central Hospital, Zhanjiang 524045, PR China; Zhanjiang Institute of Clinical Medicine, Central People's Hospital of Zhanjiang, Guangdong Medical University Zhanjiang Central Hospital, Zhanjiang 524045, PR China; Zhanjiang Institute of Clinical Medicine, Central People's Hospital of Zhanjiang, Guangdong Medical University Zhanjiang Central Hospital, Zhanjiang 524045, PR China; Pediatric Intensive Care Unit Central, People's Hospital of Zhanjiang, Guangdong Medical University Zhanjiang Central Hospital, Zhanjiang 524045, PR China; Department of Orthopedics, Central People's Hospital of Zhanjiang, Guangdong Medical University Zhanjiang Central Hospital, Zhanjiang 524045, PR China; Zhanjiang Institute of Clinical Medicine, Central People's Hospital of Zhanjiang, Guangdong Medical University Zhanjiang Central Hospital, Zhanjiang 524045, PR China; Zhanjiang Institute of Clinical Medicine, Central People's Hospital of Zhanjiang, Guangdong Medical University Zhanjiang Central Hospital, Zhanjiang 524045, PR China

## Abstract

Defects in cilia genes, which are critical for cilia formation and function, can cause complicated ciliopathy syndromes involving multiple organs and tissues; however, the underlying regulatory mechanisms of the networks of cilia genes in ciliopathies remain enigmatic. Herein, we have uncovered the genome-wide redistribution of accessible chromatin regions and extensive alterations of expression of cilia genes during Ellis–van Creveld syndrome (EVC) ciliopathy pathogenesis. Mechanistically, the distinct EVC ciliopathy-activated accessible regions (CAAs) are shown to positively regulate robust changes in flanking cilia genes, which are a key requirement for cilia transcription in response to developmental signals. Moreover, a single transcription factor, ETS1, can be recruited to CAAs, leading to prominent chromatin accessibility reconstruction in EVC ciliopathy patients. In zebrafish, the collapse of CAAs driven by *ets1* suppression subsequently causes defective cilia proteins, resulting in body curvature and pericardial oedema. Our results depict a dynamic landscape of chromatin accessibility in EVC ciliopathy patients, and uncover an insightful role for ETS1 in controlling the global transcriptional program of cilia genes by reprogramming the widespread chromatin state.

## Graphical Abstract

**Graphical Abstract:**
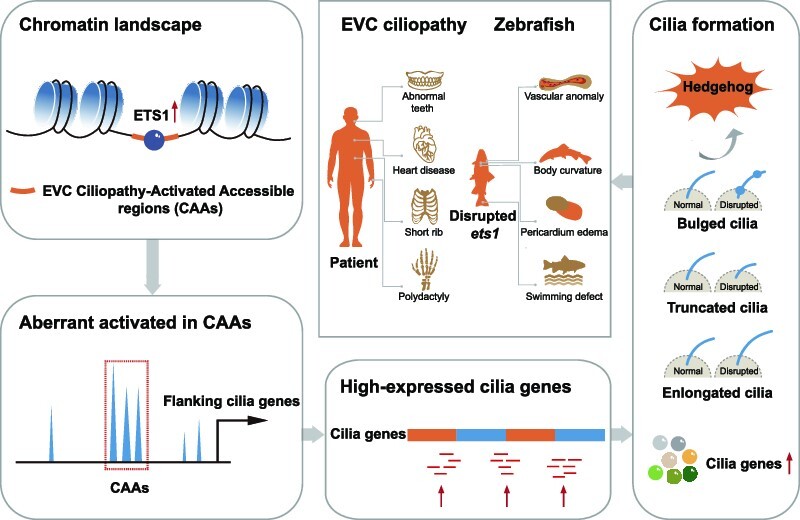

## INTRODUCTION

Ciliopathies are a group of multiorgan disorders whose aetiologies lie with primary ciliary dysfunction, leading to an expanding spectrum of diverse developmental and degenerative diseases (1,2). Although individual ciliopathy diagnoses are rare, their total contribution to human disease burden is surprisingly equal to that of several more common diseases, with an estimated incidence of ∼1:1000 (3). To date, >20 phenotypically distinguishable symptoms have been associated with ciliopathies. Although individual diseases are known for the most commonly affected organ, for each of these ciliopathies remarkable phenotypic variability has been observed even between members of the same family, making clinical diagnosis a challenge. For example, Ellis–van Creveld syndrome (EVC) is one of the skeletal ciliopathies that not only exhibits the general clinical symptoms of ciliopathies, such as polydactyly, characteristic skeletal features, congenital cardiopathy, and developmental and intellectual delays, but may also involve unique symptoms, such as dental anomalies and ectodermal dysplasia (dysplastic nails and hair abnormalities) (4). Emerging studies have identified numerous cilia genes involving primary cilia impairment that may explain EVC ciliopathy pathogenesis, indicating that ciliopathies share common pathway defects in which the cilia act as a nexus (5).

Cilia, whose dysfunction results in ciliopathies, are microtubule-based structures located on the surface of most vertebrate cells. The assembly and disassembly of cilia rely on rigorous transcriptional programs that integrate cooperatively to direct cilia and tissue formation and development (6–8). Maintenance of cilia formation and function involves extensive diversity in gene expression and dynamic interaction between the cilia regulation axis and multimeric protein complexes that impact the downstream Hedgehog (HH), Notch and Wingless-Int (WNT) signalling pathways (9–12). Therefore, these cilia genes are crucial to human development, especially when considering their collective contributions to the development of ciliopathies. To date, hundreds of cilia genes have been implicated in established ciliopathies; meanwhile, potentially hundreds more cilia genes associated with ciliary structures and/or functions could result in as yet unknown or novel ciliopathies (13). Studies based on candidate gene approaches have provided insightful views for understanding the pathogenesis of ciliopathies (14,15). Nevertheless, additional diseases have emerged that were not traditionally considered to be ciliopathies; therefore, previous research focusing on individual genes is insufficient to fully explain the transcriptional alterations that happen in ciliopathy-critical events. Conversely, very little information exists regarding the gene regulatory networks involved in ciliopathies. Therefore, to understand the molecular basis of ciliopathies, it is critical to identify the regulatory elements governing the changes in cilia gene expression.

Alterations in epigenetic landscapes are known to contribute to gene regulatory networks (16). As a substantial part of epigenetic regulation, the collaboration of chromatin accessibility sites with regulatory elements profoundly changes the chromatin state, which subsequently influences the downstream transcriptional program (17). Indeed, among the genomic factors that may affect biological behaviour at a given locus, chromatin accessibility is the most important, and pioneer factors that target closed chromatin can lead to its opening (18–20). The use of the innovative epigenetic Assay for Transposase-Accessible Chromatin with sequencing (ATAC-seq) has advanced our understanding of the machinery of epigenetic modification and gene expression regulation in processes such as tumorigenesis and embryonic development (21–23). Although previously identified genetic alterations have been recognized in ciliopathies, the heterogeneity in the specific genetic alterations between patients provides convincing evidence that epigenomic regulation is crucial to understanding ciliopathy pathogenesis. The disruption of the structure of topologically associated chromatin domains (TADs) led to abnormal limb formation through the rewiring of epigenome-mediated gene–enhancer interactions in a case of cilia-related ciliopathy (24). Epigenetic mechanisms, including DNA methylation and histone modifications, were found to be vital for cooperation between cilia genes and the regulatory network in polycystic kidney disease (PKD)-associated ciliopathies (25,26). The widespread phenotypic changes in ciliopathies indicate that extensive alterations in the gene expression programs that control cilia disassembly and signalling response must be required for the disease to develop (27). Therefore, it is imperative to understand the specific regulatory changes controlling gene expression that lead to ciliopathy pathogenesis.

In this study, we explored the global redistribution of chromatin accessibility leading to broad transcriptome effects in EVC ciliopathy patients. We observed a significant increase in accessibility sites, defined as EVC ciliopathy-activated accessibility regions (CAAs), and modulation of the kinetics of neighbouring cilia genes. Furthermore, the transcription factor (TF) ETS1 was recruited to the CAAs and acted as a fundamental regulator for global alterations of the chromatin state. Suppression of ETS1 reduced the activity of CAAs and disturbed the expression program of cilia genes. Consistent with the phenotype of ciliopathy patients, disruption of *ets1* expression in zebrafish caused body curvature and heart dysfunction. Collectively, our findings reveal the role of ETS1-driven chromatin accessibility redistribution in the regulatory network modulation of critical cilia genes, indicating a novel appendage-patterning pathway previously unrecognized in EVC ciliopathy pathogenesis.

## MATERIALS AND METHODS

### Human material and cell culture

Venous blood was collected using standard procedures under ethical approval of the Institutional Ethics Committee of the Central People's Hospital of Zhanjiang (KY-YS-2021–05), with informed consent obtained from the patients and healthy donors. The peripheral blood mononuclear cells (PBMCs) were isolated using Ficoll (Cytiva, 17544202, USA) according to the manufacturer's instructions.

hTERT RPE-1 cells (ATCC, CRL-4000, USA) were cultured in F12/Dulbecco’s modified Eagle’s medium (DMEM) (1:1) supplemented with 10% foetal bovine serum (GeminiBio, 900-108, USA) and 0.01 mg/ml hygromycin B under 5% CO_2_ at 37°C. To facilitate ciliation, hTERT RPE-1 cells were serum starved in DMEM/F12 (1:1) supplemented with 0.01 mg/ml hygromycin B for 24–48 h.

### Lentivirus preparation and transfection

The second-generation packaging system plasmids pMD2.G and psPAX2 (Addgene, USA) were used to create lentivirus in 293T cells. Transfection was performed on 5 × 10^6^ 293T cells growing in a 10 cm culture dish using polyethylenimine (PEI) with 4 μg of lentiviral vector, 3 μg of psPAX2 and 1 μg of pMD2.G (lentiviral vector:psPAX2:pMD2.G = 4:3:1). Cells were collected every 24 h between 24 h and 72 h post-transfection, ultracentrifugation was used to concentrate the supernatants, and the virus titre was determined through successive dilutions.

### Antibodies

All antibodies are listed in the Supplementary Data.

### ATAC-seq and data processing

Fresh isolated PBMCs and hTERT RPE-1 cells were harvested and resuspended with cold lysis buffer for 10 min on ice. Next, 50 000 nuclei were counted using trypan blue staining and prepared for transposition reactions according to the manufacturer's instructions for the TruePrep DNA Library Prep Kit V2 for Illumina (Vazyme, TD501, China). Subsequently, nuclei were pelleted and resuspended with transposase at 37°C for 30 min. DNA fragments were then purified with AMPure XP beads (Beckman, A63882, USA). DNA libraries were constructed after eight cycles of polymerase chain reaction (PCR) amplification using the TruePrep DNA Library Prep Kit and sequenced on the Novaseq PE150 platform to a depth of 4.0 × 10^7^ reads.

For ATAC-seq data processing, FastQC (version 0.11.7) was used to evaluate the quality of the sequencing data, and Trimmomatic was explored to remove the adaptor sequences and obtain the clean data, which were subsequently aligned to the hg38 reference genome using Burrows–Wheeler alignment (BWA). Multiply mapped reads were removed using SAMtools (version 1.3.1) and the MASCS2 was applied to call significant peaks with a q-value < 0.05. To identify differentially expressed genes between healthy donors and patients, the ATAC-seq peaks of each sample were merged to generate a consensus set of unique peaks. The number of peaks among this set was counted using bedtools (version 2.25.0), and CAAs and ciliopathy-inactivated accessibility regions (CIAs) were identified using DESeq2 (version 1.16.0) (28), with the thresholds |log2FC| > 1 and *P* < 0.05. Genomic features of peaks were annotated using the ChIPseeker R package. Additionally, Homer software (version 4.6) and CentriMo (version 5.4.1) were applied to discover binding motifs in CAAs and CIAs. Genome-wide normalized signal coverage tracks were created by bam Coverage in deepTools (version 3.3.0) and were visualized in the Integrative Genomics Viewer (IGV version 2.5.0).

### CUT&Tag and data processing

First, 10 000 fresh PBMCs and hTERT RPE-1 cells were counted and processed according to instructions for the Hyperactive^®^ Universal CUT&Tag Assay Kit for Illumina (Vazyme, TD903, China). Briefly, ConA beads (concanavalin A-conjugated paramagnetic beads) were added to the cell pellet and incubated at room temperature for 10 min. Subsequently, the mixed complexes were incubated overnight at 4°C with primary antibody. The cells were then washed, followed by incubation with diluted secondary antibody at room temperature for 2 h. Antibody–protein–DNA complexes were then linked by pA/G-Tnp and incubated at room temperature for 1 h. After washing, the eluted complexes were subsequently sheared by adding 5× Trueprep Tagment Buffer L (5× TTBL) provided in assay kit. Proteinase K and DNA extract beads were used for obtaining eluted DNA. Sequencing libraries were established after 11 cycles of PCR amplification. Selected sizes were processed using AMPure XP beads and the DNAs were sequenced on the Novaseq PE150 platform to a depth of 2.5 × 10^7^ reads. For CUT&Tag data processing, FASTQC was used to measure the data quality distribution, and the clean reads were then aligned to reference genome sequences using the bwa program. Peaks were called using MACS2 peak caller with q-value < 0.05. Genome-wide normalized signal coverage tracks were visualized in the IGV. Peaks that were >2-fold changed at a false discovery rate (FDR) < 0.1 were considered differential peaks, while peaks that did not have a log2 fold change (FC) significantly different from zero were termed constitutive peaks.

### ChIP-qPCR analyses

PBMCs were cross-linked with 1% formaldehyde solution (Sigma, F8775, USA) for 10 min at room temperature. Glycine was added for 5 min to quench the fixation reaction. Subsequently, the chromatin was sheared into 300–500 bp fragments using Bioruptor^®^. The sheared DNA was pre-cleared by adding 20 μl of salmon sperm DNA/Protein A Sepharose beads (Sigma, GE17-5280, USA) and incubated at 4°C for 30 min. Next, 3 μg of primary antibody was incubated with a 250 μg fraction of beads–DNA mixture and incubated overnight at 4°C. The following day, antibody–protein–DNA complexes were pulled down using 30 μl of Protein A Sepharose bead slurry. After washing, the eluted complexes were subsequently de-cross-linked at 65°C for 5 h. Finally, the ChIP (chromatin immunoprecipitation) DNA was purified using phenol–chloroform extraction for further quantitative PCR (qPCR) study. The primers used in ChIP-qPCR are listed in the Supplementary Data.

### Immunofluorescence and microscopy

In brief, PBMCs and hTERT RPE-1 cells for immunofluorescence were seeded on coverslips in 100 mm culture dishes. At 70–80% confluence, cells were washed twice with 1× ice-cold phosphate-buffered saline (PBS) and fixed with 4% paraformaldehyde at room temperature for 20 min. Subsequently, the cells were blocked with 1% bovine serum albumin (BSA) at room temperature for 30 min and incubated overnight at 4°C diluted in 1% BSA with primary antibody. Subsequently, the cells were washed and incubated with 150 μl of secondary antibody at room temperature for 2 h. 4′,6-Diamidino-2-phenylindole (DAPI) stain was added with a final concentration of 2 ng/μl, and staining time was limited to 3 min. Finally, slides were sealed with 5 μl Fluoromount-G^®^ mounting medium, and observed under a fluorescence microscope after 5 h.

All the centrosomal and cilia proteins were observed at room temperature using a TH4-200 fluorescence microscope (Olympus, Japan) equipped with a ×60,  1.42 numerical aperture (NA) Apo oil objective lens (Olympus, Japan) or an A1-SHR LFOV confocal microscope (Nikon, Japan) equipped with a ×60 , 1.42 NA and ×100 , 1.4 NA objective lens (Nikon). Images were acquired using DP Controller software (Olympus, Japan) or NIS-Elements software (Nikon, Japan). For images observed using z-stacking, sections were acquired with 0.5 μm distance between z-steps. All images were reconstructed to maximum projections. Images were processed using ImageJ and Photoshop (Adobe, USA).

### Cilia measurement analyses

The Pythagorean theorem (PyT) was used to achieve the optimal type of measurement of cilia anatomy/structure in 3D space. Knowing the length of two sides in a right-angle triangle, the third side can be calculated using the PyT formula: *a*^2^ + *b*^2^ = *c*^2^. To calculate the precise cilium length *c*, the formula can be rewritten as: *c* = (*a*^2^ + *b*^2^)^$\frac{1}{2}$^, where the length of the cilium on the maximum intensity projection is *a*, and the thickness of z-slices is *b* (29).

### Expression analysis

For western blotting, the total protein was isolated using TRIzol reagent (Invitrogen, 10296028, USA) according to the manufacturer's protocol. The proteins were dissolved in 1% sodium dodecylsulphate (SDS), separated using SDS–polyacrylamide gel electrophoresis (PAGE), and transferred to a nitrocellulose membrane. The membranes were pre-stained using Ponceau S buffer and blocked in 5% BSA for 1 h. The tailored membranes were incubated overnight at 4°C with the primary antibodies. After washing, the membranes were incubated with a diluted secondary antibody for at least 2 h at room temperature, washed three times with PBST, and finally visualized using the Odyssey infrared imaging system (Odyssey, USA).

For total RNA analysis and data processing, the total RNA was extracted using TRIzol reagent according to standard protocols, followed by analysis using an Agilent 2100 BioAnalyzer (Agilent Technologies, USA) to verify RNA integrity. Library construction and high-throughput RNA-seq were performed with the Novaseq PE150 platform to a depth of 1.5 × 10^7^ reads. The sequencing adaptors were removed from the raw RNA-seq reads, and the obtained clean data were aligned to the human reference genome (hg38) using HISAT2 software (version 2.1.0.). For gene expression quantification, the sequencing reads within each gene were counted, and the counts were normalized using edgeR (30). Enriched Gene Ontology (GO) terms were identified with MGI Gene Ontology Term Finder using |log2FC| > 1 and *P*-value < 0.05 as thresholds.

### RNA interference (RNAi)

For the knockdown experiment, two verified small interfering RNAs (siRNAs) of ETS1 (31) from GenePharma were transfected into hTERT RPE-1 cells. The cells were analysed 48–72 h after siRNA transfection. To obtain siRNA-resistant ETS1 (resETS1), silent mutations were incorporated into the siRNA-targeted ETS1 sequence. All sequences are provided in the Supplementary Data.

### Zebrafish maintenance and microinjections

Zebrafish experiments were performed under ethical approval of the Institutional Ethics Committee of the Central People's Hospital of Zhanjiang (ZJDY2022-13). Adult zebrafish stocks of the AB strain were maintained under standard aquaculture conditions. All embryos were incubated at 28.5°C.

Morpholinos were designed and synthesized by Gene Tools, LLC (Philomath, USA), including a standard control morpholino and a morpholino targeting the start codon region (AUG) of *ets1* transcripts to block translation. Morpholino oligonucleotides of 2 ng/embryo of control or *ets1* were separately injected into the yolk at the one-cell stage. Morpholino sequences are provided in the Supplementary Data.

The *ets1* coding sequence (CDS) was ligated to the PCS2 + vector at the BamH$\iota$ site through Gibson assembly. *ets1* mRNA was *in vitro* transcribed using the mMESSAGE mMACHINE SP6 kit (Invitrogen, AM1340, USA). For overexpression and rescue, 200 pg of *ets1* mRNA was injected into each embryo at the one-cell stage.

### Whole-mount *in situ* hybridization (WISH)

The *cmlc2* RNA probe was *in vitro* transcribed and digoxigenin labelled using the DIG RNA Labeling Kit (Roche, 11277073910). WISH was performed as reported (32).

### Whole-mount immunofluorescence staining for zebrafish embryo

Embryos were fixed at the 8-somite stage using 4% paraformaldehyde at 4°C overnight. Anti-acetyl-alpha-tubulin antibody (1:500, Sigma, MABT868, USA) was used to label the cilia in Kupffer's vesicles (KVs). Immunofluorescence staining was performed as previously described (33). Before permeation with acetone, embryos were equilibrated in Tris–HCl (0.1 M, pH 9.0) for 5 min at room temperature and then heated at 70°C for 15 min for antigen retrieval. After staining, embryos were flattened and imaged using the z-stack function of the confocal microscope with a ×60 oil objective.

### Behavioural assays

The behavioural assay was performed using a ZebraBox system and video tracking software (ViewPoint Life Sciences, France). At 5 days post-fertilization (dpf), an individual larva was placed in each well of a 96-well plate containing 500  μl of embryo medium without light exposure. Plates were sealed with an optical adhesive film to prevent evaporation. The movement of each larva was recorded over a period of 20 min. A transparent background mode with a detection threshold of 20 was set. Behavioural endpoints were swimming distance (cm) and time (s).

### Heart failure assessment

The zebrafish larvae were subjected to video recording under a Zebralab Blood Flow System (ViewPoint Life Sciences, France). To determine differential atrial and ventricular heart rate, heart videos were analysed using MicroZebraLab (ViewPoint Life Sciences). The pixel density changes detected by the software correlate with cardiac muscle contractions and chamber filling, which are used to estimate cardiac muscle contractions in beats per minute (bpm).

Blood flow videos were analysed using ZebraBlood (ViewPoint Life Sciences). The pixel density changes detected by the software are combined with vessel diameter to determine the flow rate for each frame. Quantitative assessments were performed using video-based analysis, from which the linear velocity, blood flow and vessel diameter were evaluated.

### Statistical analysis

Either a two-tailed unpaired Student's *t*-test or a one-way analysis of variance (ANOVA) with Dunnett's multiple comparisons test was applied to determine the difference between groups, unless otherwise noted, using GraphPad Prism 5 (version 5.01; GraphPad, USA). The results are presented as the mean ± standard error of the mean (SEM), and *P* < 0.05 was considered to indicate a statistically significant difference, unless otherwise stated.

## RESULTS

### Open chromatin landscape undergoes distinct global remodelling in EVC ciliopathy patients

**Figure 1.**
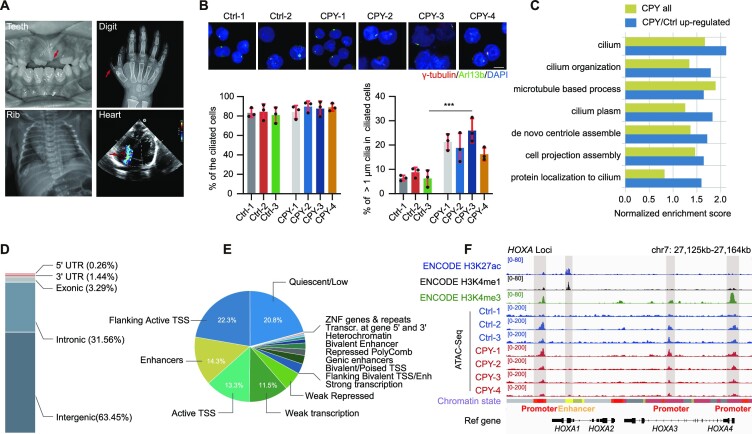
Open chromatin landscape remodelling occurs in EVC ciliopathy patients. (**A**) Oral, skeletal and radiological results displaying the characteristics of EVC ciliopathy. (**B**) Immunostaining of γ-tubulin (red) and Arl13b (green) in PBMCs of normal individuals (Ctrl) and EVC-associated ciliopathy patients (CPY). DNA was stained with DAPI (blue) (upper). Quantification of the percentage of ciliated cells (lower left) and cilia > 1 μm in ciliated cells (lower right). ***P < 0.001, as determined using Student's *t*-test. Scale bars, 2 μm. (**C**) Cilia-related GO term enrichment scores within EVC ciliopathy and up-regulated EVC ciliopathy genes from the transcriptome dataset. (**D**) Annotations of the genomic distribution of all peaks identified using ATAC-seq. UTR, untranslated region. (**E**) Annotations of all open chromatin regions presenting the chromatin states trained using public data of PBMCs from the ENCODE project. TSS, transcription start site. (**F**) The IGV snapshot displaying the H3K27ac, H3K4me1 and H3K4me3 signals of PBMCs from ENCODE, and ATAC-seq signals of PBMCs in *HOXA* gene loci. Vertical grey boxes indicate enhancer and promoter ATAC-seq signals. Chromatin states are obtained from ENCODE (red, active promoter; yellow, weak enhancer; orange, strong enhancer; green, transcribed region; and grey, heterochromatin).

One of the common malformations associated with ciliopathies is post-axial polydactyly. Here, we recruited patients with polydactyly predominantly of the hands and/or of the feet; based on previous reports and sequencing information, four of these patients were clinically diagnosed with the EVC ciliopathy, due to the additional presence of chondrodysplasia (dental abnormalities, short rib), congenital cardiopathy, intellectual disability and variable degrees of ectodermal dysplasia (dysplastic nails, hair abnormalities) (Figure 1A; Supplementary Data). Since cilia defects are responsible for the phenotypes in ciliopathies, and since it has been reported that PBMCs can be ciliated (34), we first examined the cilia morphology of PBMCs isolated from the patients. We found that the cilia in the PBMCs of these patients were longer than those in normal individuals, demonstrating the structural cilia defects in the patients, which strengthens the correlation between cilia malfunction in patient-derived cells and the symptoms observed in the patients (Figure 1B). Additionally, the transcriptional expression levels of several well-recognized cilia genes in the PBMCs, including *CEP131*, *NEK8* and other cilia genes recorded in the SCGSv2 gold standard list, were substantially elevated in the genome-wide transcriptome profile of EVC ciliopathy patients (35) (Supplementary Figure S1A–C). Notably, the differentially expressed genes were always enriched in multiple signalling processes—such as body morphogenesis, metabolic processes and immune responses—that have been shown to be closely associated with cilia genes (36–39) (Supplementary Figure S1D, E). In particular, the genes overexpressed in patients were always enriched in cilia-related pathways, reinforcing that a common set of cilia genes were activated during EVC ciliopathy pathogenesis (Figure 1C).

Chromatin accessibility is a reflection of and corresponds to the transcriptome profile that determines the cellular state (40). Given the unique alterations in cilia genes and the enrichment of cilia-related pathways, we conducted ATAC-seq analyses in isolated PBMCs from both patients and healthy donors (Supplementary Figure S2A). As expected, the ATAC-seq data showed clear nucleosome phasing in the insert size distributions, and the reads were appropriately enriched at transcription start sites (TSSs) (Supplementary Figure S2B, C). Peaks representing the chromatin accessibility regions were primarily located in the intergenic regions containing abundant *cis*-regulatory elements; furthermore, genome annotation revealed that a large fraction of these ATAC-seq peaks overlapped with promoter and enhancer regions, consistent with previous findings (41) (Figure 1D, E). Additionally, the gene expression profile alterations always coincided with changes in promoter accessibility, reinforcing the idea that the ATAC-seq-detected accessible regions co-localize extensively with promoter elements (Supplementary Figure S2D). After comparing our ATAC-seq data with public data from the Encyclopedia of DNA Elements (ENCODE), a large fraction of our ATAC-seq peaks overlapped with the signals in promoters marked by the histone modification H3K4me3 (Supplementary Figure S3A, B). For instance, the *HOXA* and *PTCH1* genes involved in regulating limb morphology exhibited strong ATAC-seq H3K4me3 signals in the promoters, but not enhancer signals marked by H3K4me1 and H3K27ac (42,43) (Figure 1F; Supplementary Figure S2E), indicating promoter occupancy of the chromatin accessibility signal in EVC ciliopathy.

### Identification of specific chromatin accessible regions in EVC ciliopathy patients

**Figure 2.**
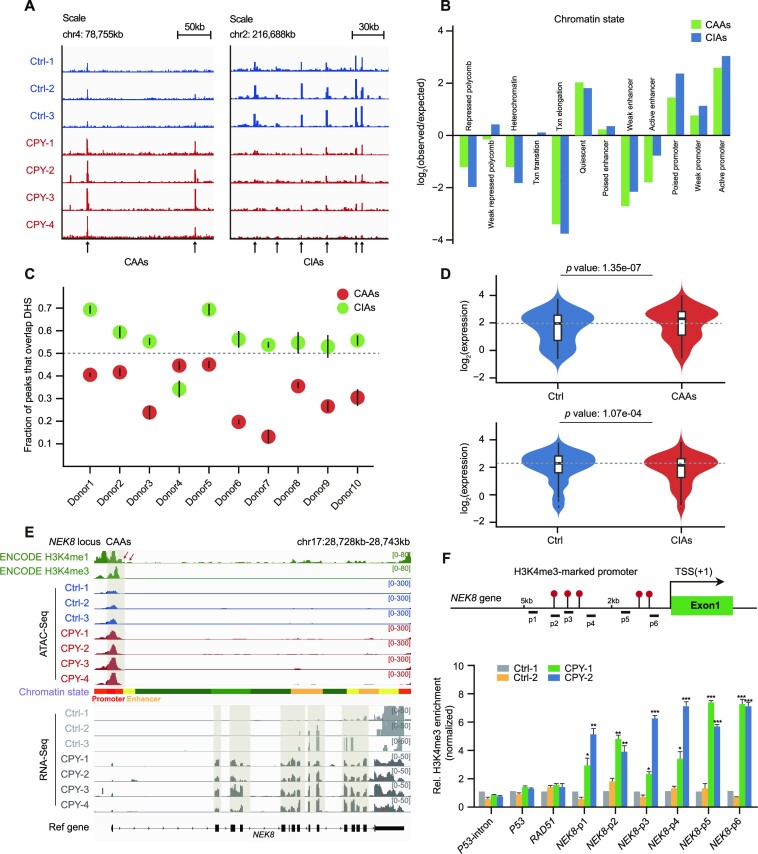
Genome-wide identification and characterization of EVC ciliopathy-related DARs. (**A**) Insertion tracks of EVC CAAs and EVC CIAs at chromosome 4 (left) and chromosome 2 (right) loci. Differentially open regions are marked with arrows. (**B**) The defined enrichment of chromatin states at CAAs and CIAs. (**C**) Overlap of CAAs and CIAs with DNase-hypersensitive sites (DHSs) of PBMCs from 10 healthy donors. Mean overlap with DHS peak calls is shown. Bars represent 95% confidence intervals. (**D**) Violin plots displaying the distributions of expression changes in CAA- (upper) and CIA-adjacent (lower) genes. (**E**) Snapshot displaying the H3K4me1 and H3K4me3 signals of PBMCs from ENCODE, and the ATAC-seq and RNA-seq signals of PBMCs in the *NEK8* gene locus. Vertical grey boxes indicate the promoter region ATAC-seq signals and corresponding *NEK8* gene expression. (**F**) Location diagram of H3K4me3 ChIP-qPCR primers in the *NEK8* locus (upper). The relative levels of H3K4me3 for CAAs in the *NEK8* gene measured using ChIP-qPCR in PBMCs (lower). H3K4me3 enrichment for the *P53* intron served as a negative control, and H3K4me3 enrichment for *P53* and *RAD51* served as positive controls, as previously described. The enrichment is normalized to 10% input, while IgG is used as a negative control. Error bars represent the means ± SEM for three independent experiments. *P < 0.05, **P < 0.01, ***P < 0.001, as determined using one-way ANOVA with Dunnett's multiple comparisons test.

To identify chromatin remodelling that potentially contributes to EVC ciliopathy pathogenesis, we defined >10 000 differentially accessible regions (DARs) between EVC ciliopathy patients and healthy donors based on quantitative peak signals; these DARs included 402 EVC CAAs and 568 EVC CIAs (Figure 2A; Supplementary Figure S4A, B). As expected, promoter elements were enriched in both CAAs and CIAs according to chromatin state analysis (Figure 2B). Furthermore, most CAAs and CIAs were located ∼2 kb from TSSs, indicating the mapping of the potential promoter elements in the peaks mentioned above (Supplementary Figure S4C–E). Using ChIP-qPCR of H3K4me3, randomly selected peaks in CAAs and CIAs were validated based on their promoter signal alterations (Supplementary Figure 4F). By comparing our data with public DNase-seq data from ENCODE, we found that >50% of the peaks in CIAs overlapped with the chromatin open regions in data from healthy donors, whereas the fractions of overlapped peaks in CAAs were substantially lower in all healthy donors, representing the unique accessibility pattern of CAAs in patients (Figure 2C).

### CAAs regulate adjacent cilia gene expression

Because promoters have the ability to act as enhancers to regulate the expression of nearby and distal genes in cell fate decision (26), we investigated the impact of EVC ciliopathy-specific DARs on global gene expression. Interestingly, CAA-neighbouring genes were up-regulated, while those adjacent to CIAs were down-regulated (Figure 2D). Additionally, genes known to be up-regulated in patients, such as *NEK8*, showed substantial elevations in both chromatin accessibility and expression level (Figure 2E). Verification of the H3K4me3 signal near *NEK8* underscored the previous evidence for increased promoter activity at CAAs (Figure 2F).

**Figure 3.**
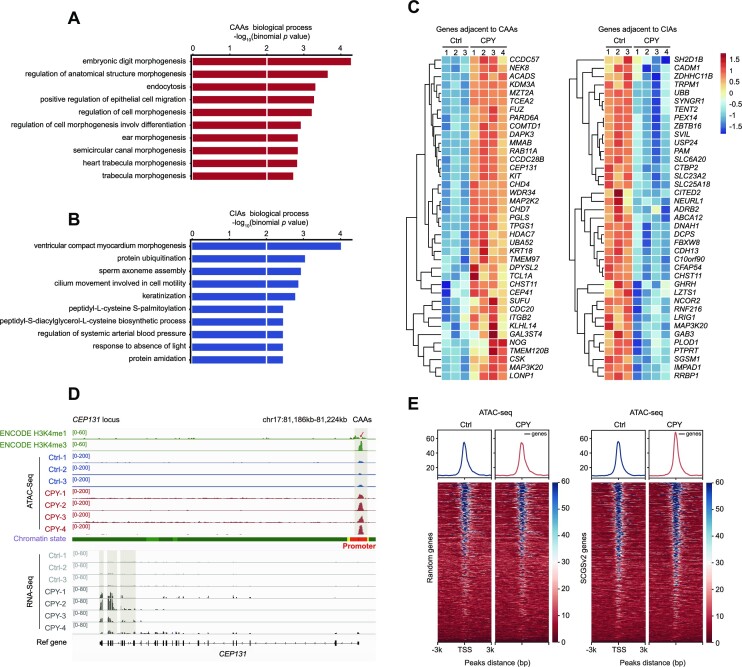
CAAs affect expression profiles of CAA-adjacent cilia genes. (**A** and **B**) GO enrichments of (A) CAAs and (B) CIAs using Genomic Regions Enrichment of Annotations Tool (GREAT) analysis. Bar length represents the enriched *P*-value for biological processes. (**C**) Heatmaps displaying normalized gene expression among controls and patients. Genes adjacent to CAAs (left panel) and CIAs (right panel) are listed. (**D**) Snapshot displaying the H3K4me1 and H3K4me3 signals of PBMCs from ENCODE, and the ATAC-seq and RNA-seq profiles of PBMCs at the representative cilia gene *CEP131*. Vertical grey boxes indicate promoter region ATAC-seq signals and corresponding *CEP131* gene expression. (**E**) Heatmaps and enrichment plots showing normalized read densities of ATAC-seq signals for randomly selected genes (*n* = 700, left) and SCGSv2 genes (right) in Ctrl and CPY at the TSS. Tracks are centred at the TSS, extending ± 3 kb.

Next, GO analysis was used to identify the biological functions of DAR-adjacent genes. Notably, CAA-adjacent regions were enriched in morphogenesis-associated pathways, including embryonic digit morphogenesis, cell morphogenesis and morphogenesis of other organs, which are crucial for the development process (Figure 3A). Meanwhile, genes neighbouring CIAs were predominantly enriched in metabolism-associated pathways such as protein ubiquitination and keratinization (Figure 3B). Consistent with the GO analysis results, CAA-adjacent genes were significantly up-regulated, including the well-known cilia genes *CEP131*, *CEP41* and *CDC20* (Figure 3C, D; Supplementary Figure S5A, B). Conversely, *RNF216*, the key regulator of protein ubiquitination in development, was flanked by CIAs and showed decreased expression in patients (Figure 3C; Supplementary Figure S5C). Since we showed that the genes neighbouring the CAAs always displayed up-regulated expression, and that many of these aberrant elevated CAA-adjacent genes were well-known cilia genes, we wondered whether these cilia genes had a regulatory role in chromatin accessibility dynamics. Therefore, we quantified the ATAC-seq intensities from the gold standard SCGSv2 list, and found that peaks in patients led to substantial enrichment of cilia genes compared with the signal in healthy donors, while the randomly selected gene set displayed unchanged signals in patients and healthy donors (Figure 3E). Collectively, our results suggest that the CAA-harbouring EVC ciliopathy accompanied by multiorgan developmental abnormalities is due to the impaired cellular signalling; moreover, the newly activated CAAs are tightly coupled to a robust increase in the expression of adjacent cilia genes, which may serve as a prerequisite or contributing factor for the aberrant elevated cilia gene expression seen in patients.

### Recruitment of ETS1 to CAAs contributes to regulation of the expression of cilia genes

**Figure 4.**
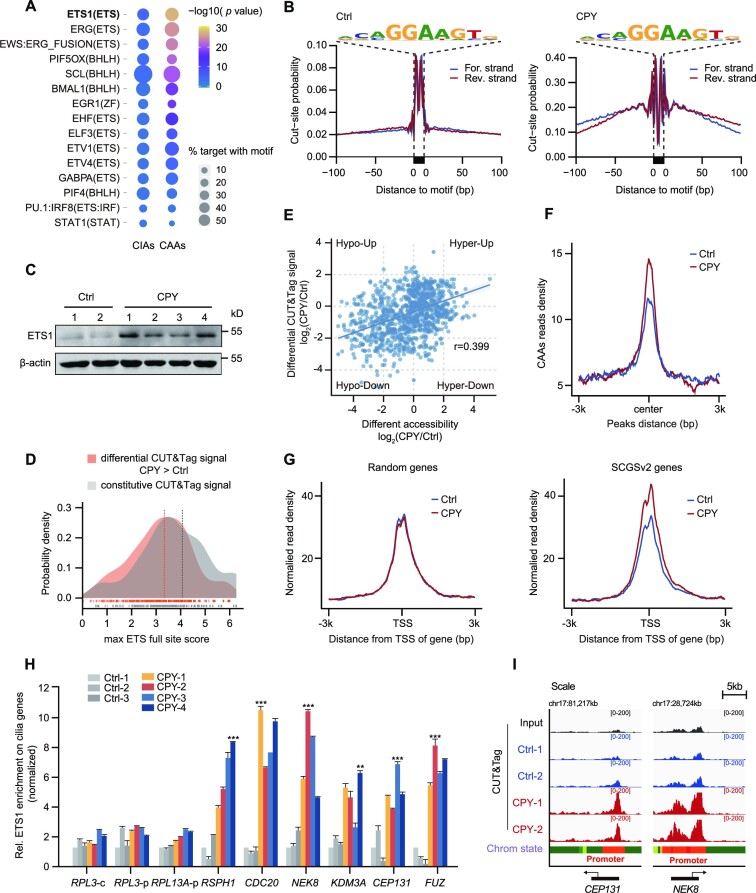
ETS1 is recruited to CAAs and leads to increased expression of cilia genes. (**A**) Top-ranked enriched motifs among CAAs and CIAs are listed, as determined using HOMER2 algorithms. The circle size represents the percentage of motifs in the target regions, and the colour represents the *P*-value. (**B**) ATAC-seq footprint at the ETS motif site in controls and patients. Insertions per site are normalized to have the same average number of insertions 200–500 bp away from the motif. (**C**) Western blotting analysis of total ETS1 protein expression in Ctrl and CPY. β-Actin was used as a loading control. (**D**) Distribution of motif scores of sites within ETS1 CUT&Tag peaks in PBMCs, either those that gave more signal in CPY than in the Ctrl (differential) or those that were not significantly different (constitutive). The maximum scoring ETS full site within each CUT&Tag peak was used. The constitutive peaks have a higher mean motif score than the differential peaks (Mann–Whitney U-test, *P* < 1 × 10^−32^). (**E**) Spearman's correlation of differential ETS1 CUT&Tag signals in accessible regions and differential accessibility in CPY versus Ctrl (*r* = 0.399). (**F**) The enrichment of ETS1 CUT&Tag signals at CAAs in both Ctrl and CPY. Tracks are centred at the peaks and extend ± 3 kb. (**G**) Enrichment plots showing normalized read densities of ETS1 CUT&Tag signals at the TSS for randomly selected genes (*n* = 700, left) and SCGSv2 genes (right) in both Ctrl and CPY. Tracks are centred at the TSS, extending ± 3 kb. (**H**) The relative levels of cilia genes flanking CAAs in Ctrl and CPY measured using ETS1 ChIP-qPCR in PBMCs. ETS1 enrichment for *RPL3*-c (coding region) served as the negative control, and ETS1 enrichment for *RPL3*-p (promoter region) and *RPL13A*-p (promoter region) served as positive controls, as previously described. The enrichment of ETS1 is normalized to 10% input. **P < 0.01, ***P < 0.001, as determined using one-way ANOVA with Dunnett's multiple comparisons test. (**I**) IGV snapshot showing the ETS1 CUT&Tag signals of PBMCs in Ctrl and CPY at chromosome 17 loci of cilia genes *CEP131* and *NEK8*.

To identify the potential TFs involved in chromatin remodelling at CAAs, thus contributing to the regulation of downstream cilia genes, we carried out motif enrichment analyses using the algorithms HOMER and MEME. The top-ranked motifs were observed in the consensus binding sites for ETS, bHLH, ZF and the STAT family, but only the ETS motif was strongly over-represented in CAAs (Figure 4A; Supplementary Figure S6A, B). Therefore, we carried out footprinting analyses and found considerable TF occupancy around the ETS motif site in EVC ciliopathy patients relative to healthy donors, suggesting a protracted ETS–DNA interaction on CAAs during EVC ciliopathy pathogenesis (Figure 4B). The ETS family of proteins comprises 28 TFs that contain a highly conserved DNA‐binding ETS domain (44) (Supplementary Figure S6C). To identify putative TFs that regulate CAAs, we subsequently screened the ETS TFs exhibiting expression changes and found that ETS1 expression—both for mRNA and for protein—was notably higher in EVC ciliopathy patients relative to healthy donors (Figure 4C; Supplementary Figure S6D, E).

To further determine whether ETS1 maintains the chromatin landscapes, we assessed ETS1 occupancy in PBMCs from both EVC ciliopathy patients and healthy donors by using CUT&Tag. Notably, ETS1 displayed a genome-wide signal increase in EVC ciliopathy patients relative to healthy donors (Supplementary Figure S6F). We quantified and compared the motif scores of the CUT&Tag peaks of controls and EVC ciliopathy patients, and found that, on average, peaks that were differentially occupied had lower motif scores than peaks that were constitutively occupied, which is consistent with the finding that increased ETS1 expression enables binding of lower affinity sites (Figure 4D). Moreover, the differential accessibility and CUT&Tag signal were relatively correlated, indicating that a high fraction of differential accessibility is attributable to differential ETS1 occupancy (Figure 4E). In particular, the ETS1 enrichment signal on CAAs was elevated in EVC ciliopathy patients relative to that in healthy individuals, reinforcing the potential function of ETS1 in modulating the activity of CAAs (Figure 4F).

Since we have revealed that CAAs are responsible for robust downstream cilia gene expression, ETS1 enrichment among randomly selected genes was compared with the signal around cilia genes in the SCGSv2 gold standard list. Indeed, these cilia genes exhibited pronounced ETS1 enrichment flanking their TSSs relative to a randomly selected gene set (Figure 4G). Moreover, ChIP-qPCR of ETS1 was performed to examine its signal on CAAs flanking cilia genes, which showed that the signal strength of ETS1 was substantially increased on these CAAs (Figure 4H). IGV mapping of ETS1 occupancy on *CEP131* and *NEK8* also emphasized the role of ETS1 in recruitment to this subset of CAAs (Figure 4I). Altogether, these results suggest that the recruitment of ETS1 to CAAs substantially altered the chromatin state transitions, ultimately causing downstream transcriptional alterations associated with increased expression of cilia genes.

### ETS1 is required for cilia formation

**Figure 5.**
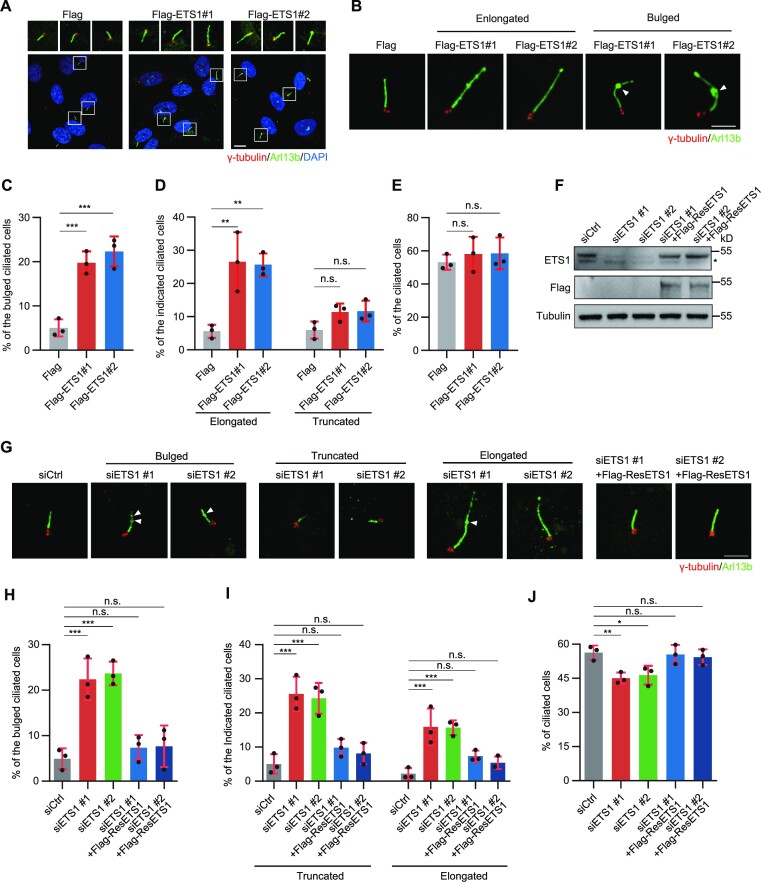
ETS1 positively regulates cilia formation. (**A**) Immunostaining of γ-tubulin (red) and Arl13b (green) in ETS1-overexpressing hTERT RPE-1 cells. DNA was stained with DAPI (blue). Scale bars, 5 μm. (**B**) Immunostaining of γ-tubulin (red) and Arl13b (green) in ETS1-overexpressing hTERT RPE-1 cells. Arrowheads indicate the bulge of cilia. Scale bars, 2 μm (**C–E**) Quantification of the percentage of bulged (C), elongated (left) or truncated (right) (D), and total (E) ciliated cells, from (A) and (B). (**F**) Immunoblots of ETS1 and Flag expression in control (siCtrl), ETS1-depleted (siETS1) and ETS1-rescued (siETS1 + Flag-ResETS1) hTERT RPE-1 cells. Tubulin was used as a loading control. An asterisk indicates non-specific ETS1 bands. (**G**) Immunostaining of γ-tubulin (red) and Arl13b (green) in control, ETS1-depleted and ETS1-rescued hTERT RPE-1 cells. DNA was stained with DAPI (blue). Arrowheads indicate the bulge of cilia. Scale bars, 2 μm (**H–J**) Quantification of the percentage of bulged (H), truncated (left) or elongated (right) (I), and total (J) ciliated cells from (G). For C, D, E, H, I and J, error bars represent the means ± SEM for three independent experiments. n.s., not significant, *P < 0.05, **P < 0.01, ***P < 0.001, as determined using one-way ANOVA.

Previous studies have hypothesized the potential function of genes from the ETS family in regulating the expression of cilia genes during tumour progression (45). As a member of the ETS family, ETS1 is suspected to be an oncogene involved in various cancers and to have a vital function in haematopoietic cell development (46,47). To further investigate the potential role of ETS1 in EVC ciliopathy, we performed ETS1 overexpression and knockdown experiments in hTERT RPE-1 cells and observed the impact on cilia formation after serum starvation. It was demonstrated that forced ETS1 expression resulted in severe cilia morphology impairments including bulge, truncation (< 3 μm) and elongation (> 8 μm), with a higher percentage of elongated than truncated cells (Figure 5A–D), although the increase in the overall percentage of ciliated cells was insignificant (Figure 5A, E). Conversely, it was found that ETS1 knockdown led to almost the opposite effects of its overexpression. ETS1 knockdown impaired cilia morphology with remarkably increased bulged cilia, a higher percentage of cells with truncated cilia than elongated cilia and a slight increase in the overall percentage of cells that underwent cilia formation. These morphology impairments, along with the quantity of ciliated cells, were rescued by treatment with exogenous siRNA-resistant ETS1 (Figure 5F–J; Supplementary Figure S6G). Collectively, these results revealed the critical and unexpectedly bidirectional regulator role of ETS1 in cilia formation.

### Disrupted chromatin accessibility caused by ETS1 silencing underlies the impaired transcriptional program of cilia genes

**Figure 6.**
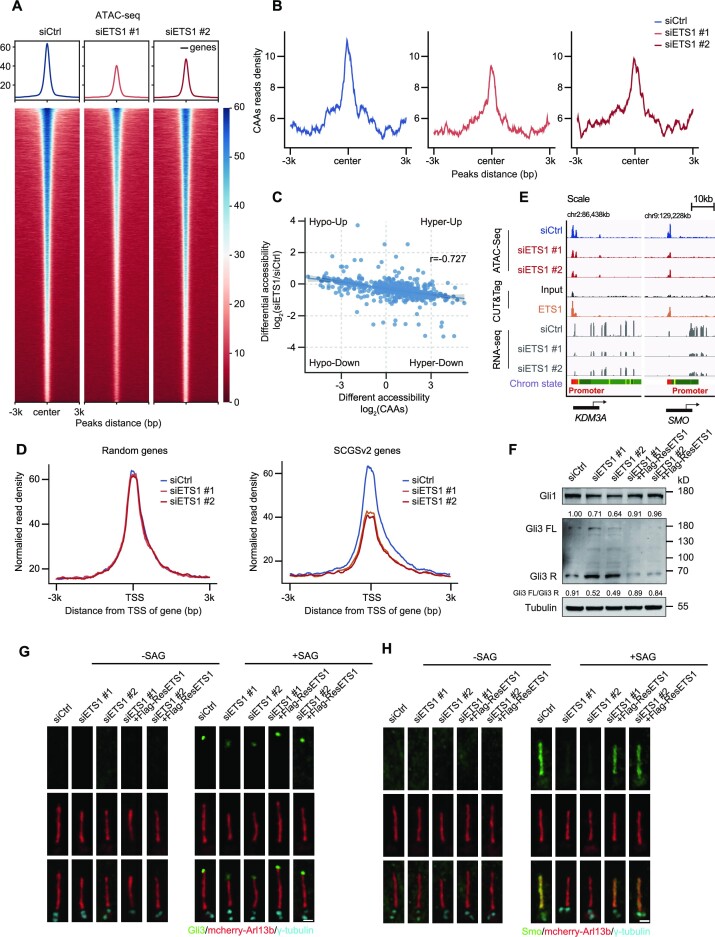
ETS1 loss induces aberrant expression of cilia genes by remodelling the signals of flanking CAAs. (**A**) Heatmaps and enrichment plots showing normalized read densities of ATAC-seq signals after down-regulation of ETS1 expression in hTERT RPE-1 cells. Tracks are centred at the peaks and extend ± 3 kb. (**B**) The enrichment of ATAC-seq signals at CAAs after down-regulation of ETS1 expression in control (siCtrl) and ETS1-depleted (siETS1) hTERT RPE-1 cells. Tracks are centred at the peaks and extend ± 3 kb. (**C**) Spearman's correlation of CAA accessibility changes and regions that close after ETS1 suppression in hTERT RPE-1 cells (*r* = –0.727). (**D**) Enrichment plots showing normalized read densities of ATAC-seq signals from hTERT RPE-1 cells at the TSS for randomly selected genes (*n* = 700, left) and SCGSv2 genes (right) after ETS1 knockdown. Tracks are centred at the TSS and extend ± 3 kb. (**E**) IGV snapshot showing the ATAC-seq, CUT&Tag and RNA-seq signals from hTERT RPE-1 cells in loci of *KDM3A* and *SMO* genes. (**F**) Immunoblots of Gli1 and Gli3 in control (siCtrl), ETS1-depleted (siETS1) and ETS1-rescued (siETS1 + Flag-ResETS1) hTERT RPE-1 cells. The intensity of Gli1 and the ratio of Gli3-FL/Gli3-R was quantified. Tubulin was used as a loading control. (**G** and **H**) Immunostaining of Gli3 (green) (G) or Smo (green) (H) and γ-tubulin (cyan) in control, ETS1-depleted and ETS1-rescued hTERT RPE-1 cells transfected with mCherry–Arl13b (red) with (right) or without (left) Smoothened agonist (SAG). Scale bars, 1 μm.

To determine if prolonged ETS1 expression is required to sustain an open chromatin state of CAAs, we performed a stable ETS1 knockdown in hTERT RPE-1 cells; using ATAC-seq on siETS1 samples and negative controls, we found that ETS1 silencing caused extensive alterations in chromatin accessibility (Supplementary Figure S7A). The peak annotation suggested that the closure of accessibility in ETS1 knockdown cells occurred primarily in intergenic regions enriched in ETS-family motif sites (Supplementary Figure S7B, C). Notably, ETS1 knockdown substantially reduced accessibility, revealing an ETS1-driven disruption of the global open chromatin state, supporting the view that ETS1 is capable of inducing chromatin remodelling by evicting nucleosomes directly from nucleosome-bound DNA (48) (Figure 6A). In contrast to the elevated CAA peaks mentioned above, the suppressed expression of ETS1 resulted in a robust decrease of CAA activity (Figure 6B). Meanwhile, considering that nucleosomes and TFs are in competition for DNA-binding sites, we evaluated nucleosome occupancy after ETS1 knockdown by calculating the ATAC-seq data insert sizes. We observed an increase in nucleosome occupancy at ETS motif sites in ETS1-silenced cells, leading to less ETS1 occupancy on CAAs (Supplementary Figure S7D). Additionally, the closed chromatin regions in ETS1 knockdown cells were generally anti-correlated with accessibility changes in CAAs, highlighting the function of ETS1 in restructuring chromatin accessibility during EVC ciliopathy pathogenesis (Figure 6C).

Given that the SCGSv2 genes exhibited substantially decreased densities after ETS1 silencing relative to randomly selected genes, we then tested ETS1 occupancy in hTERT RPE-1 cells by using CUT&Tag techniques (Figure 6D; Supplementary Figure S7E). In ETS1 knockdown cells that always displayed ETS1 occupancy, we observed the collapse of CAA activity in regions including the well-described cilia genes *KDM3A* and *SMO*, further supporting the role of ETS1 in harbouring CAAs that flank cilia genes (49,50) (Figure 6E). To verify whether ETS1 alone can sufficiently regulate the expression of cilia genes, we carried out transcriptome profiling in ETS1 knockdown cells and found extensively decreased expression of cilia genes adjacent to CAAs corresponding to ETS1 silencing (Supplementary Figure S7F, G). Gene set enrichment analysis (GSEA) of the transcriptome profiling revealed that many cilia-related signalling pathways, including the HH pathway, were impaired after ETS1 depletion (Supplementary Figure S7H–J). This finding inspired us to investigate whether ETS1 is required for ciliary signalling transduction. GLI Family Zinc Finger 3 (Gli3), a critical TF in HH signalling, exists in two forms: a full-length activator (Gli3-FL) or a repressor (Gli3-R). Gli3-FL activates the HH pathway and modulates HH genes by targeting the Gli1 promoter, whereas Gli3-R—produced by degradation of Gli3-FL—inhibits HH functions. In this study, Gli3-R expression decreased and Gli1 expression substantially increased in Smoothened (SMO) agonist (SAG)-treated ETS1-overexpressing cells, indicating that forced ETS1 expression activated the HH signalling pathway (Supplementary Figure S8A). Furthermore, depletion of ETS1 inhibited the HH signalling pathway, and exhibited the opposite response in Gli1 and Gli3 expression patterns compared with ETS1 overexpression (Figure 6F). These results demonstrated that ETS1 plays a critically positive role in the HH signalling pathway.

Consistently, SAG-treated ETS1-overexpressing cells exhibited activated HH signalling with a notable increase in Gli3 accumulation at the cilia tip (Supplementary Figure S8B, C). Meanwhile, SAG-induced ciliary SMO localization was apparently enhanced in ETS1-overexpressing cells (Supplementary Figure S8D, E). Conversely, depletion of ETS1 inhibited HH signalling transduction with impaired Gli3 accumulation at the cilia tip and weakened SMO localization along the cilia (Figure 6G, H; Supplementary Figure S8F, G). Therefore, these phenomena further confirmed that ETS1 is a positive regulator of the canonical HH signalling pathway. Altogether, our data support a EVC ciliopathy model in which ETS1 is recruited to CAAs to stabilize the open chromatin state, thereby inducing a genome-wide program of expression of cilia genes related to cilia-associated processes such as signalling transduction.

### ETS1 is sufficient for cilia development in zebrafish larvae

**Figure 7.**
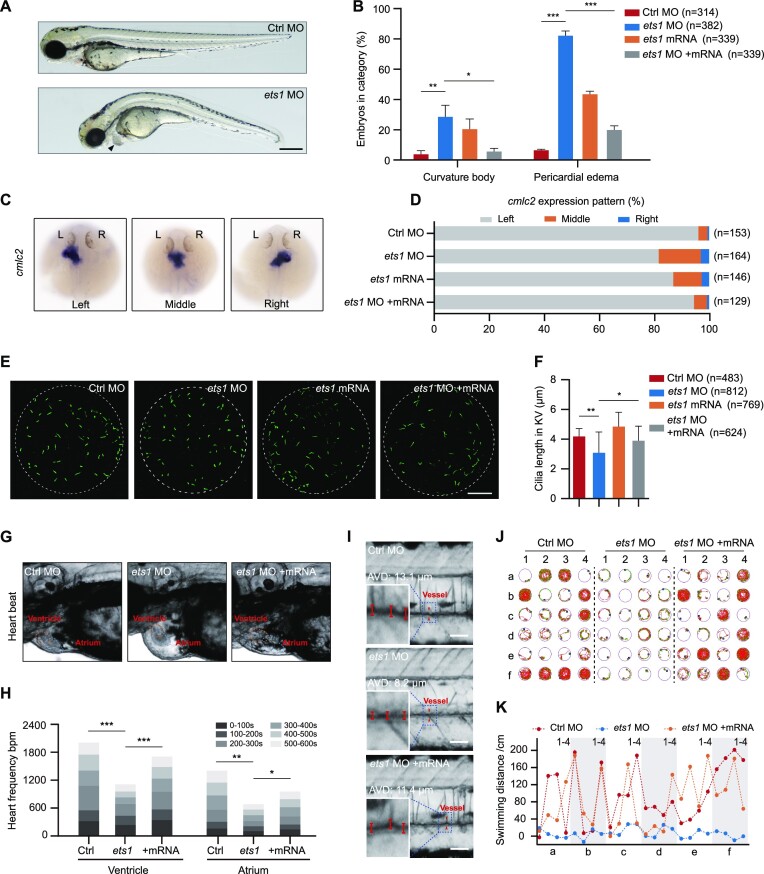
ETS1 expression alteration results in multiple ciliary defects in zebrafish larvae. (**A**) Representative image of *ets1* morphants (*ets1* MO, bottom) in bright field showed pericardial oedema and body curvature phenotypes. The arrowhead indicates pericardial oedema. Scale bar, 500 μm. (**B**) Percentage of embryos exhibiting body curvature and pericardial oedema phenotypes in control, *ets1* morphants, *ets1*-overexpressing (*ets1* mRNA) and *ets1*-rescued (*ets1* MO + mRNA) zebrafish larvae. *P < 0.05, **P < 0.01, ***P < 0.001, as determined using Student's *t*-test. (**C** and **D**) WISH results (C) and corresponding statistics (D) of *cmlc2* at 30 hpf displayed deficiencies in left–right asymmetry after *ets1* knockdown and overexpression. (**E** and **F**) Immunofluorescence staining using anti-acetyl-alpha-tubulin antibody at the 8-somite stage (E) and corresponding quantification (F) displayed decreased cilia length in the KV of *ets1* morphants. *P < 0.05, **P < 0.01, as determined using Student's *t*-test. Scale bar, 20 μm. (**G** and **H**) The heart rate, expressed in bpm, in the ventricle and atrium over various time intervals for a total time of 10 min. The ventricle and atrium in control, *ets1* morphants and *ets1* mRNA-rescued larvae are labelled with dashed circles (G). Quantification of the results is shown in (H). *P < 0.05, **P < 0.01, ***P < 0.001, as determined using Student's *t*-test. (**I**) The representative blood flow dynamics map of control, *ets1* morphants and *ets1* mRNA-rescued larvae at 5 dpf. The linear flow meter process was captured in a movie to show the change of blood flow in real time. AVD, average vessel diameter. Scale bar, 100 μm. (**J** and **K)** Representative results of locomotion trajectories (J) and swimming distance analysis (K) in control, *ets1* morphants and *ets1* mRNA-rescued larvae at 5 dpf during a total time of 20 min. The green line represents the trajectory at moderate velocity, and the red line represents the trajectory at high velocity.

ETS1-driven redistribution of transcriptome profiling in cilia genes in patients provides an important context for understanding organ development. Here, we evaluated these developmental changes in zebrafish, a powerful model system for the study of cilia structure, and examined the changes in cilia formation and subsequent morphological and physiological effects caused by alterations in ETS1 expression. A morpholino targeting the ‘AUG’ site of the *ets1* transcript was designed and synthesized (Supplementary Figure S9A). At 72 hours post-fertilization (hpf), *ets1* morphants displayed various typical cilia defect phenotypes, including body curvature (29.1%) and pericardial oedema (82.6%). Intriguingly, overexpression of ETS1 also led to these cilia defect phenotypes, but with lower frequency (body curvature, 20.8%; pericardial oedema, 43.9%) (Figure 7A, B). Meanwhile, we performed WISH using a *cmlc2* probe to examine heart position distribution. In *ets1* morphants, 15.2% and 3.1% of embryos displayed middle and right-sided heart, respectively. In line with the morphogenetic phenotype results, ETS1-overexpressing embryos also displayed left–right asymmetry defects; however, only 3.3% and 0.7% of the control embryos displayed middle and right-sided heart, respectively (Figure 7C, D). These results imply that *ets1* plays a vital role in determining left–right patterning. Notably, an increased percentage of truncated cilia compared with the control group could be detected not only in ETS1-deficent cells but also in ETS1-overexpressing cells, although with a lesser effect. Thus, we observed cilia defect phenotypes—including body curvature, pericardial oedema and disorder of left–right asymmetry—in both *ets1* morphants and ETS1-overexpressing zebrafish. Moreover, we note that both depletion and overexpression of a protein called nuclear distribution gene C (NudC) that regulates ciliogenesis can lead to the above-mentioned cilia defect phenotypes in zebrafish (51), indicating that both excessive and insufficient expression of cilia-related genes in zebrafish can result in the occurrence of cilia defect phenotypes, and that the precise expression of cilia genes is critical for zebrafish development.

In zebrafish, left–right asymmetry patterning is regulated by the ciliated organ the KV (52). Hence, we also labelled the cilia in the KV at the 8-somite stage, measured the length of the cilia and discovered that those of *ets1* morphants were significantly shorter than those of control embryos (Figure 7E, F). To validate the specificity of phenotypes in *ets1* morphants, we co-injected a mixture of *in vitro* transcribed *ets1* mRNA with *ets1* morpholino. Notably, all of the defects caused by *ets1* morpholino, including body curvature, pericardial oedema, disruption of left–right asymmetry, and cilia length defects in the KV, were effectively rescued by exogenous *ets1* mRNA (Figure 7B–F). These results rule out morpholino-induced toxicity or secondary effects, and validate the specificity of the phenotypes in *ets1* morphants.

Since ETS1 has been shown to have an essential function in angiogenesis, we speculated that heart pumping capacity and blood flow would change after ETS1 repression or overexpression, which may lead to pericardial oedema in zebrafish (53). Hence, we measured the heart rate in zebrafish larvae over a 10 min period and found that the atrial and ventricular beat rates were robustly reduced in *ets1* morphants relative to controls, whereas rescuing *ets1* in the *ets1* morphants partially restored the heart rates toward the levels of the controls (Figure 7G, H). As expected, in larvae overexpressing *ets1*, heart rates were decreased relative to controls (Supplementary Figure S9B, C). These findings suggest that a specific level of *ets1* gene expression is necessary for heart development. Cardiac morphology is one of the important indicators to evaluate cardiac function, which affects systemic blood circulation. To further address the impact of ETS1 on blood circulation, we quantified the blood flow dynamics by evaluating linear velocity, blood flow and vessel diameter, and discovered severe vascular stenosis in *ets1* morphants and larvae overexpressing *ets1* (Figure 7I; Supplementary Figure S9D–F). Moreover, restoring *ets1* expression with mRNA in *ets1* morphants ameliorated the symptoms of vascular stenosis (Figure 7I; Supplementary Figure S9D). This finding explains the heart pumping defects induced by either ETS1 repression or overexpression. Swimming is a highly complex behaviour that involves deeply integrated physiological processes, and organ defects are known to profoundly affect exercise ability. Therefore, we analysed zebrafish locomotor trajectories to further assess behavioural changes in response to alterations in ETS1 expression. During a 20 min test, we noticed that the ETS1-suppressed and ETS1-overexpressing zebrafish had substantially decreased swimming distance and swimming time relative to control zebrafish, while rescue of *ets1* expression with mRNA in zebrafish with *ets1* knockdown resulted in increased swimming distance and swimming time relative to zebrafish with ETS1 repression (Figure 7J, K; Supplementary Figure S9G, H). In summary, our results provide evidence that ETS1 functions as the important regulator in organ development, the malfunction of which negatively affects the fins and heart, in line with the characteristics of ciliopathies.

## DISCUSSION

A myriad of cilia genes involved in cilia formation and function profoundly affect cilia signalling transduction, leading to changes in physiological and developmental functions. Deciphering the cilia gene expression program within complex networks and pathways is crucial for understanding the underlying mechanism of multiorgan-associated ciliopathies. Nevertheless, knowledge of the effects of cilia gene regulation on ciliopathies is currently limited; therefore, it is vital to thoroughly characterize the chromatin state-dependent mechanism for the cilia regulatory network. In this study, we investigated the dramatic remodelling of the chromatin state in EVC ciliopathy patients. The distinct CAAs were activated by a single TF, ETS1, leading to further up-regulation of the expression of flanking cilia genes. We also confirmed aberrant ETS1 activation in EVC ciliopathy patients, while suppression of the ETS1-induced open chromatin state collapsed, leading to cilia defects in both cellular and organismal contexts. These data suggest the strong likelihood that cilia genes require ETS1-induced chromatin state alterations as their mechanism for EVC ciliopathy pathogenesis; moreover, these findings underscore the powerful combination of genome-wide epigenetic approaches with individual molecular studies to uncover previously unknown variations in rare diseases.

ETS-family TFs have been well characterized in several types of solid tumours. Although their role in ciliopathies has not been studied, a recent study attempted to establish the link between ETS-family TFs and modulation of cilia gene expression at the tumour–microenvironment (TME) interface (45). Our findings resolve the question of the mechanism by which ETS1 drives the redistribution of chromatin accessibility, thereby directing the regulatory network of critical cilia genes in EVC ciliopathy patients, coinciding with a previous study showing that ETS1 can maintain the function of cilia by binding to the IFT20 promoter to facilitate its expression (54). In our study, the ETS1-regulated ciliary genes can be primarily divided into two groups based on their effects on cilia: the ciliary positive factors that form the majority of ETS1-regulated genes, and include *WDR34*, *RSPH1*, *CEP41*, *NEK8*, *CEP131* and *FUZ*, and the ciliary negative factors that include only a few genes responsible for ciliary disassembly, such as *CDC20* and *KDM3A*. Therefore, it is reasonable that the ETS1-overexpressing cells exhibited more elongated cilia than truncated cilia, thus creating a net positive effect including the slightly increased cilia formation and activation of HH signalling seen in ETS1-overexpressing cells, with a different but not completely opposite effect on cilia in ETS1-deficient cells. Therefore, unlike most unidirectional regulation of cilia in single gene defects, ETS1 demonstrates bidirectional regulation in cilia structure and function. Notably and consistently, continuously high ETS1 expression in EVC ciliopathy patients will lead to the aberrant activation of cilia genes; however, lack of ETS1 expression leads to failure to open accessible regions that regulate correct expression of cilia genes, implying the essential role of ETS1 for development.

Usually, polydactyly and bone defects are related to defective HH signalling. Notably, ectopic expression of Shh in the anterior limb bud also causes the formation of extra anterior digits (55). Previous studies have reported that ETS/ETV-family TFs regulate Shh expression at E11.5 during the maintenance/expansion phase of Shh expression, and these studies also indicate that ETS1 activates HH signalling (56,57). Moreover, a recent study has found that overexpressed ETV2 can displace the chromatin of limb enhancers that ectopically activate Shh signalling and induce polydactyly (58). Thus, it is reasonable that EVC ciliopathy patients who have enhanced HH signalling induced by ETS1 overexpression exhibit the symptom of polydactyly.

Additionally, there are now well-supported experimental distinctions between confirmed and suspected cilia/ciliopathy genes, primary and motile cilia genes, and cilia genes that encode proteins that are active only within cilia and those that encode proteins that are active both within and outside of cilia. In our study, for example, many of these screened cilia genes function within cilia, such as CEP131 and CEP41, while others function both within and outside of cilia, such as FUZ—which governs trafficking of intraflagellar transport (IFT) proteins from the cytoplasm to the basal body and then to the ciliary tip (59)—and KDM3A, which regulates recruitment of IFT proteins into the cilia by modulating actin dynamics through actin cytoskeleton binding (49). Collectively, these results indicate the comprehensive modulation of the transcriptional program of cilia genes by ETS1.

Apart from ETS1, it is well demonstrated that RFX and FOXJ1 TFs directly regulate genes for core ciliary components. RFX TFs play essential roles in the generation of both motile and sensory cilia. The RFX proteins activate core components such as IFT122, IFT172, Dync2li1, Dnah9 and Dnah11, thereby regulating a series of ciliary genes that positively regulate cilia formation and function. Meanwhile, FOXJ1 programs motile cilia by activating a network of motile cilia genes that promote cilia formation and function, for different cilia types, in selected cell types and organisms. However, the TFs of the Forkhead (FOX) and RFX families either could not be recruited by accessible chromatin regions or were not activated in our TF–CAAs–cilia genes axis (Supplementary Figure S10A–C). In contrast to RFX proteins and FOXJ1, ETS1 activates two groups of cilia genes that can positively and negatively modulate cilia formation and function, leading to bidirectional phenotypes very different from those generated by the ciliogenesis ‘master regulator’ TFs, including RFX proteins and FOXJ1. Notably, manipulation of ETS1 expression in different cell types may produce distinct cilia phenotypes, paralleling species-specific differences in the ability of TFs such as FOXJ1 to regulate cilia.

Recent research shows that ETS-family TFs can serve as transcriptional activators and/or repressors according to gene and context, revealing the complicated multidirectional function of ETS-family TFs in cilia regulation (45). Whether and to what extent the other ETS TFs function in cilia modulation, and how these family members precisely orchestrate cilia formation and function requires further elucidation. In addition to the ETS family, the TFs of other families may also participate in the control of expression of cilia genes, which will probably further validate the great importance of chromatin dynamics and epigenetics in ciliopathies (14,60).

Based on previous findings, the ETS family members are oncogenic TFs connected to wide-ranging cellular processes such as self-renewal, DNA damage, metabolism, TME modulation, dynamic chromatin remodelling and epigenetics (61); furthermore, cilia also take part in these processes. Recent studies have strengthened the genetic and functional links between the DNA damage response (DDR) and ciliary factors including CEP63, CEP152, CEP164, CEP290, MCPH1, NEK8 and PCNT (62). Additionally, primary cilium, as a spatially localized platform for various signal transduction pathways, plays a crucial role in the TME, since paracellular signalling between tumour cells and other cells in the TME strongly affects initiation, progression and therapeutic efficacy in tumours (63). The signal transduction, mediated by primary cilium, also participates in energy and steroid metabolism (64), which affects tumour cell survival. Recently, the function of ETS-family TFs in modulating cilia gene expression at the TME interface has been partially elucidated (45). We have demonstrated that ETS1 regulates the expression of cilia genes via dynamic chromatin remodelling epigenetics. Combining our results with the previously reported close relationship among the ETS family, cilia and tumour formation, it is plausible that the modulation of ETS1 oncogenic efficacy is accomplished, at least in part, by primary cilia, which provides new insight into the mechanism for the oncogenic efficacy of ETS TFs, which needs further verification.

Moreover, the signalling pathway via primary cilium may also in turn affect the activity of the ETS TFs, such as FLI1. Meanwhile, Wnt/β-catenin signalling, primarily mediated by primary cilia, antagonizes the EWS–FLI1-dependent repression of transforming growth factor (TGF)-β receptor type 2 in Ewing sarcoma cells (65). Therefore, we speculate that there is a feedback loop between the ETS family and primary cilia and that this feedback loop is the means by which cells accomplish the precise regulation of cilia structure to ensure cilia function.

## Supplementary Material

gkad506_Supplemental_File

## Data Availability

All related sequencing data have been uploaded to NCBI’s Gene Expression Omnibus and are accessible through accession number GSE209662.
