# Supplementary material for: Recruitment of transcription factor ETS1 to activated accessible regions promotes the transcriptional program of cilia genes
Source: Nucleic Acids Res. 2023 Jun 16;51(13):6684–701. doi: 10.1093/nar/gkad506 (PMC10359609; doi:10.1093/nar/gkad506)
Supplement: gkad506_Supplemental_File [file gkad506_supplemental_file.pdf]

Recruitment of transcription factor ETS1 to activated accessible regions promotes transcriptional program of cilia genes

1. SUPPLEMENTARY TABLE

Clinical description

Features	CPY-1	CPY-2	CPY-3	CPY-4
Gender	F	F	M	F
Age	3	2	3	4
Operation history	N	N	N	N
Genetic history	N	N	N	N
ICF	Signed	Signed	Signed	Signed
Polydactyly	Y	Y	Y	Y
Congenital cardiopathy	Y	Y	Y	Y
Dental anomalies	Y	Y	Y	Y
Short frenula	Y	Y	Y	Y
Nail hypoplasia	Y	Y	Y	Y
Short rib	Y	Y	Y	Y
Wechsler Scale	BT	BT	BT	BT
Other anomalies	Corpus callosum hypoplasia; Renal pyelic dilatation	Cerebellar hypoplasia; Corpus callosum hypoplasia; Sparse hair	Cerebellar hypoplasia; Pulmonary hypoplasia	Corpus callosum, hypoplasia; Sparse hair

* F: Female; M: Male; N: No; ICF: Informed Consent Form Y: Yes; BT: Below the target

* The whole exome sequencing (WES) data of EVC ciliopathy patients can be accessed in NCBI's Gene Expression Omnibus (GSE209662)

Primers used in H3K4me3 ChIP-qPCR

Name	Primer sequence
CAAs-p-1	For: 5’-AGAAGAGGAAGACGGAAGCT-3’<br>Rev: 5’-TTTTGTAAATACCCTGAAGACAG-3’
CAAs-p-2	For: 5’-ATAGATGATGCGTTAAGGGTG-3’<br>Rev: 5’-TGAAGTGAACTAAGTTTGTGCC-3’
CAAs-p-3	For: 5’-CTCTACAGCCTCAACAGCAT-3’<br>Rev: 5’-AAACCACAGGGAGATACCAT-3’
CAAs-p-4	For: 5’-CTCTACAGCCTCAACAGCAT-3’<br>Rev: 5’-AAACCACAGGGAGATACCAT-3’
CAAs-p-5	For: 5’-GGAAATAATATGCACTCAGAAAC-3’<br>Rev: 5’-GCTGTCACCGTAAAAGAAAAC-3’
CIAs-p-1	For: 5’-AGGGTCAGTCTGGCTGGAGT-3’<br>Rev: 5’-ATGCGGTTTGAACATTAGGG-3’
CIAs-p-2	For: 5’-TCTTCCCACTGTGCTGCTGT-3’<br>Rev: 5’-TGAGGCCATCTCACCCTAAA-3’
CIAs-p-3	For: 5’-GTCTTTTGGCTTCTGTTTTG-3’<br>Rev: 5’-CTTCTTTTGTGGTTTAGTTTCC-3’
CIAs-p-4	For: 5’-TGTCCCCGAGGCTTCTCACA-3’<br>Rev: 5’-CCCTTCCCACCCTTCACCAC-3’
CIAs-p-5	For: 5’-GACGCTAATTGGTGACTGGT-3’<br>Rev: 5’-GTAATCCTGGCTGCTTTCTG-3’

Primers used in H3K4me3 ChIP-qPCR in *NEK8* locus

Name	Primer sequence	Notes
P53 intron	For: 5’-CTGTGGGTTTGGTGTGTGAG-3’<br>Rev: 5’--GTTGCAGGTTACAGGGCAAT-3’	Negative control (1)
P53	For: 5’-GGGTGGATGTGCAAAGAAGT-3’<br>Rev: 5’-GGGTGTAGATGATGGGGATG-3’	Positive control (1)
RAD51	For: 5’-TCTTCTCGAGCTTCCTCAGC-3’<br>Rev: 5’-AGCGCTCTTGTGGTTTGTTT-3’	Positive control (1)
NEK8-p1	For: 5’-GGGAAGAAAGAATAGGAGTTAGG-3’<br>Rev: 5’-ACGGTGGACATCGTGGCTAG-3’	
NEK8-p2	For: 5’-ATAACGCAAGAAGAAATGGG-3’<br>Rev: 5’-CTGTGAGCAGCAATCAAAAT-3’	
NEK8-p3	For: 5’-AGGGTTTGAAGGGTGTTAGC-3’<br>Rev: 5’-ACCCATTTCTTCTTGCGTTA-3’	
NEK8-p4	For: 5’-TAAGTCCTGGTCCTGGGGTGG-3’<br>Rev: 5’-GCGGGAGGGACAGGTAAACA-3’	
NEK8-p5	For: 5’-CAGGCAAACACGAAACCA-3’<br>Rev: 5’-GCCCACGAGTGACGCTATT-3’	
NEK8-p6	For: 5’-ACACATTAACCCCTGTGGGACTCTT-3’<br>Rev: 5’-CTCGCAAGGGTATCCAAAC-3’	

Primers used in ETS1 ChIP- qPCR

Name	Primer sequence	Notes
RPL3-c	For: 5’-CTGGCAATGTCCTGACAAAA-3’<br>Rev: 5’-CTGGCTGATCTCAAGTGCTG-3’	Negative control (2)
RPL3-p	For: 5’-ACATACCATCACGCCATCAA-3’<br>Rev: 5’-GGGCCAGATTTGGCTTTAT-3’	Positive control (2)
RPL13A-p	For: 5’-TCGACCAATGAAAACACAGG-3’<br>Rev: 5’- TGTCGCAGGGTTTCTTATCC-3’	Positive control (2)
RSPH1-p	For: 5’-ACTATCATTCCTCCATTGAACA-3’<br>Rev: 5’-TATCTTACTGCCACAGCGTC-3’	
CDC20-p	For: 5’-GAATCCTGGGCTCCCCTACT-3’<br>Rev: 5’-CCTGGCTAACACGGTGAAAC-3’	
NEK8-p	For: 5’-AGGGTTTGAAGGGTGTTAGC-3’<br>Rev: 5’-ACCCATTTCTTCTTGCGTTA-3’	
KDM3A-p	For: 5’-GGCATCCTTATCTTGCTCCT-3’<br>Rev: 5’-ATCTACCCAATCTCCCCACT-3’	
CEP131-p	For: 5’-CCTGTGGGTCTGGCTTCTAC-3’<br>Rev: 5’-TGTTTATGGCAGCGTCATTC-3’	
FUZ-p	For: 5’-TGGGCAACAAGAACAAAACT-3’<br>Rev: 5’-CCTCCCTGGTTCCACCGATT-3’	

Primers used in RT-qPCR

Name	Primer sequence
BCAR3	For: 5’-CACGGCGAAACCTTCACCTT-3’<br>Rev: 5’-GGGGTCCTGTCCATGATGT-3’
CEP131	For: 5’-TGATGCTCTTCGAGGGCAG-3’<br>Rev: 5’-GGAACTCCGGGCATTGGAT-3’
WDR34	For: 5’-ATCCGCTGGGAGACGAAAAG-3’<br>Rev: 5’-GGGCGTCCACATGATTCCT-3’
SUFU	For: 5’-CACGCCATCTACGGAGAGTG-3’<br>Rev: 5’-GTACTTGACGATAGCGGTAACC-3’
RPL12	For: 5’-AAAGCCCTCAAGGAACCACC-3’<br>Rev: 5’-GCATCTGTCGAGCAATGTTGAC-3’
RSPH1	For: 5’-TGTTGGCCCTGGAAAGTATGT -3’<br>Rev: 5’-GTCGGCTTTTTGGGGAGAGTT -3’
CDC20	For: 5’-GCACAGTTCGCGTTCGAGA-3’<br>Rev: 5’-CTGGATTTGCCAGGAGTTCGG-3’
FUZ	For: 5’-CGCCTCCTCCGAAACTTCTAT -3’<br>Rev: 5’-CAACACCAGGTAGCAAGCTCT-3’
KDM3A	For: 5’-ACACCGACGTTACCAAGAAGG-3’<br>Rev: 5’-CAGGTGACTTTCGTTCAGCTAA-3’
NEK8	For: 5’-GTCGGGTGTGACCATCAAG-3’<br>Rev: 5’-CCTGCACCATTTCATAGCCCA-3’

siRNA sequence used in knock down experiment

siETS1 #1	5′- GAAAUGAUGUCUCAAGCAUTT-3′
siETS1 #2	5′- CAGAAUGACUACUUUGCUATT -3′
silent mutations #1	5′-GAAATGATGTCTCAAGCAT-3′<br>5′-GAGATGATGTCACAGGCTC-3′
silent mutations #2	5′-CAGAATGACTACTTTGCTA-3′<br>5’-CAAAACGATTATTTCGCAA-3’

MO sequence used in zebrafish

Standard Control oligo	CCTCTTACCTCAGTTACAATTTATA
ets1 Morpholino oligo	TCATGGTCACGCATTCAAACGTACA

Antibodies

**Antibodies used in CUT&Tag**		
**Name**	**Manufacturers**	**Number**
ETS1	Cell Signaling Technology	14069
IgG	Proteintech	30000-0-AP
Anti-rabbit IgG H&L	Abcam	ab205718
Antibodies used in ChIP-qPCR		
Name	Manufacturers	Number
H3K4me3	Abcam	ab8580
**Antibodies used in IF**		
**Name**	**Manufacturers**	**Number**
Goat anti-Rabbit IgG (H+L) Secondary Antibody, Alexa Fluor 488	Invitrogen	Cat# A-11034
Goat anti-Mouse IgG (H+L) Secondary Antibody, Alexa Fluor 488	Invitrogen	Cat# A-11029
Goat anti-Rabbit IgG (H+L) Secondary Antibody, Alexa Fluor 568	Invitrogen	Cat# A-11036
Goat anti-Mouse IgG (H+L) Secondary Antibody, Alexa Fluor 568	Invitrogen	Cat# A-11031
Arl13b	Proteintech	Cat#17711-1-AP
Mouse anti-γ-tubulin	Sigma-Aldrich	Cat# T5326
anti-acetylated tubulin	Sigma-Aldrich	T7451
Gli3	Abcam	ab6050
Smo	Santa Cruz	sc-166685
**Antibodies used in WB**		
**Name**	**Manufacturers**	**Number**
IRDye 800CW anti-mouse	LI-COR	926-32210
IRDye 800CW anti-rabbit	LI-COR	92632211
ETS1	Proteintech	12118-1-AP
Gli1	Proteintech	66905-1-Ig
α-tubulin	Sigma-Aldrich	T9026
β-actin	Abcam	ab8226

2. SUPPLEMENTARY FIGURES AND LEGENDS

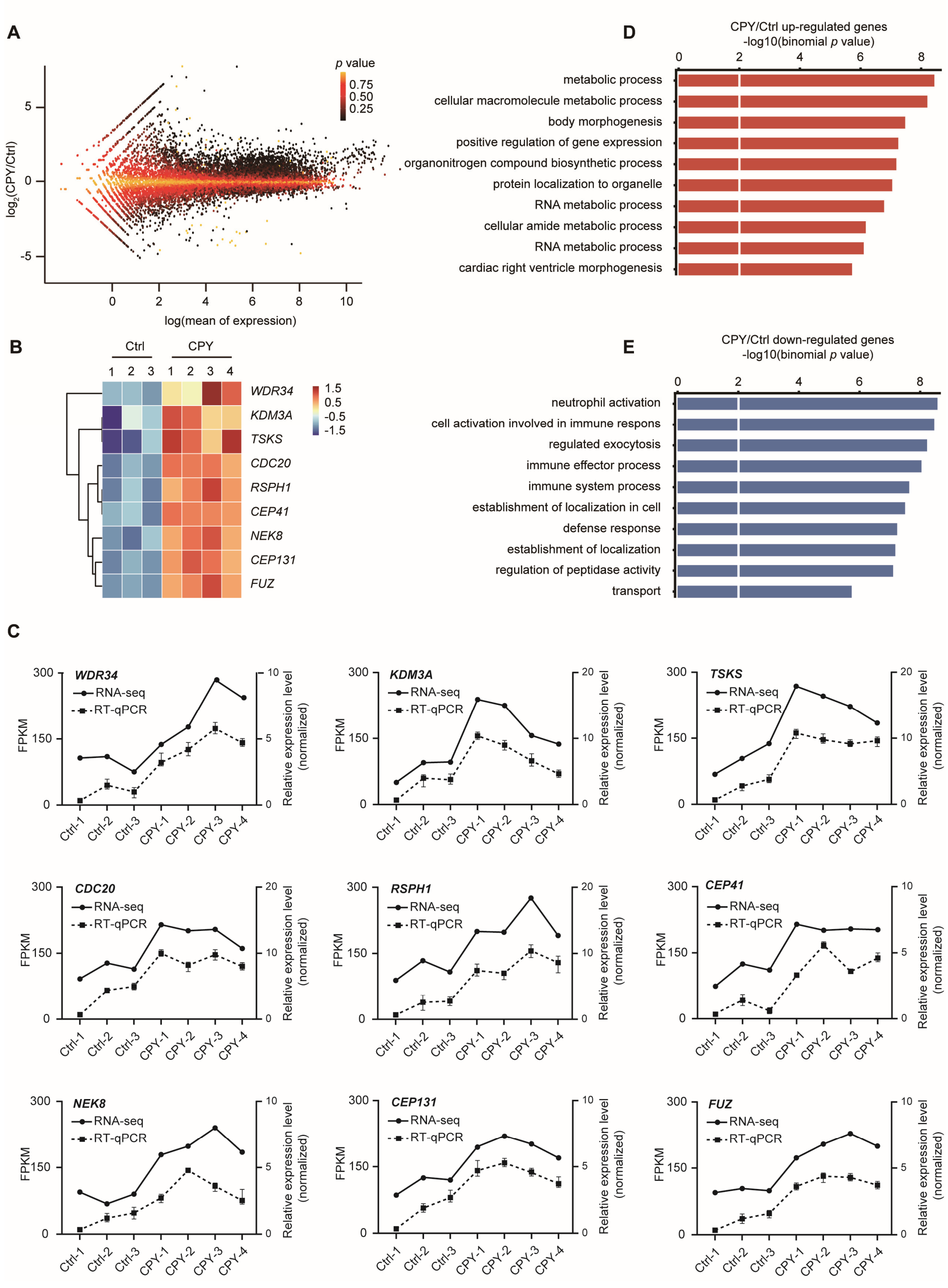

Supplementary Figure S1. Dynamics of the transcriptional program in EVC ciliopathy. (**A**) RNA-Seq scatter diagram of PBMCs showing the gene expression profiles of EVC-associated ciliopathy patients (CPY) versus healthy donors (Ctrl). (**B**) Heatmap showing representative expression of cilia genes. (**C**) The expression of *WDR34*,

KDM3A, and seven other genes in PBMCs from Ctrl and CPY. The dashed line indicates RT-qPCR results, and the solid line indicates RNA-seq data. Error bars represent the means ± S.E.M. for three independent experiments. (**D**, **E**) Gene Ontology enrichments of the (**D**) up- and (**E**) down-regulated genes in EVC ciliopathy using Genomic Regions Enrichment of Annotations Tool (GREAT) analysis. Bar length represents the enriched *p* value for biological processes.

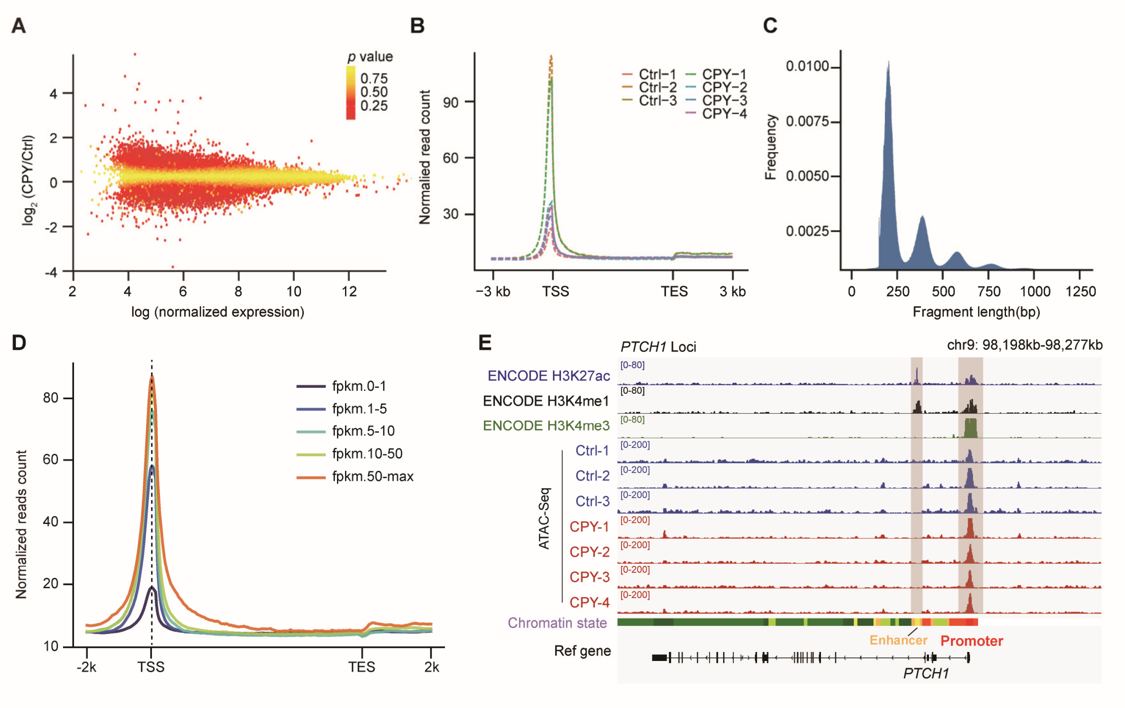

Supplementary Figure S2. Dynamics of chromatin accessibility in EVC ciliopathy. (**A**) Scatter plot of ATAC-seq signals of PBMCs showing the accessibility change of CPY and Ctrl. (**B**) The normalized read count of the ATAC-seq samples across all genes. The average ATAC-seq signals between TSS (–3 kb) and TES (+3 kb) of all genes are calculated after peak calling. TSS, transcription start site; TES, transcription end site. (**C**) Representative insert size distribution, showing clear signal modulation for mono- and di-nucleosomes. (**D**) Average ATAC-seq signals around gene bodies. Genes are divided into 5 fragments per kb of transcript (FBKM) classes according to increasing expression levels: 1 to 50 reads per million. (**E**) The Integrative Genomics Viewer (IGV) snapshot displaying the H3K27ac, H3K4me1 and H3K4me3 signals of PBMCs from ENCODE, and ATAC-seq signals of PBMCs in *PTCH1* gene locus. The vertical gray boxes indicate enhancer and promoter ATAC-seq signals. Chromatin states are obtained

from ENCODE (red, active promoter; yellow, weak enhancer; orange, strong enhancer; green, transcribed region; and gray, heterochromatin).

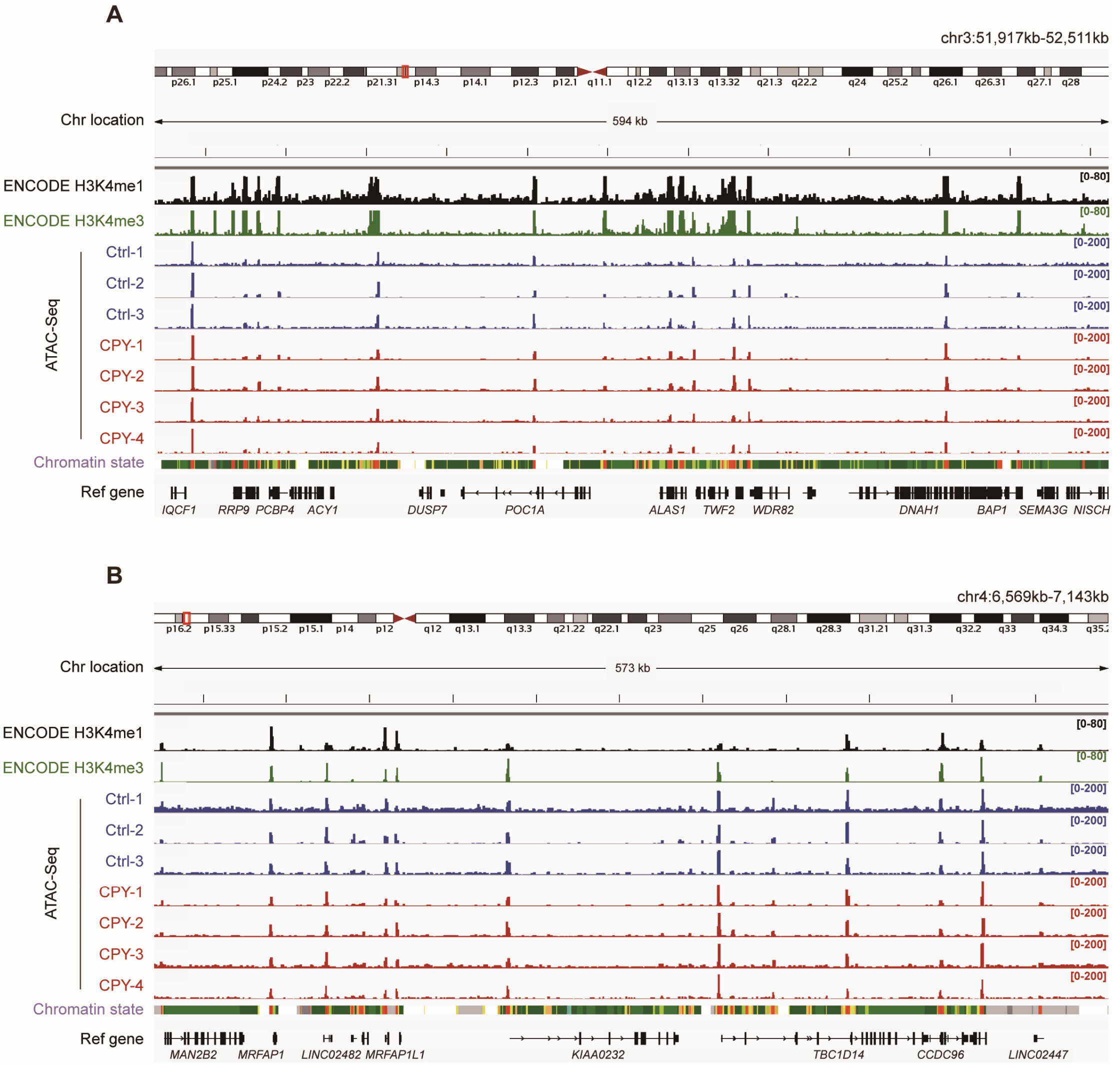

Supplementary Figure S3. ATAC-seq data shows high similarity with public Encyclopedia of DNA Elements (ENCODE) promoter data marked by histone modification H3K4me3. (**A** and **B**) Integrative Genomics Viewer (IGV) snapshot displaying the H3K4me1 and H3K4me3 signals of PBMCs from ENCODE, and ATAC-seq signals of PBMCs in randomly selected genes in (**A**) chromosome 3 and (**B**) chromosome 4.

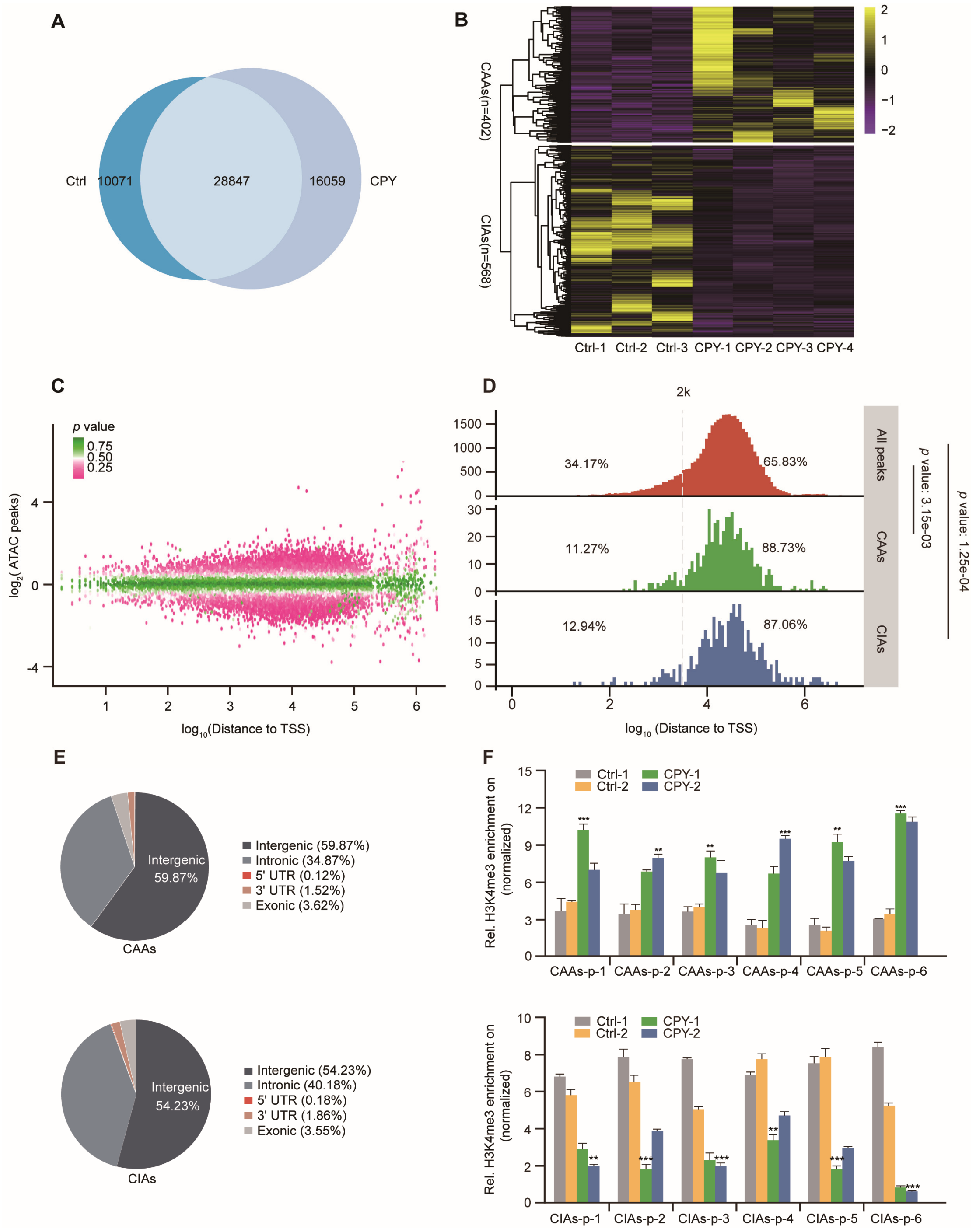

Supplementary Figure S4. Enrichment analysis of differentially accessible regions in EVC ciliopathy. (**A**) Venn diagram indicating the number of differentially accessible regions between Ctrl and CPY. (**B**) Heatmap of hierarchically clustered ATAC-seq signals of PBMCs showing the accessibility remodeling of EVC ciliopathy-activated accessible regions (CAAs) and EVC ciliopathy-inactivated accessibility regions (CIAs). (**C**) Scatter diagram showing ATAC-seq signal alterations between Ctrl and CPY in all peaks, including different transcription start site (TSS) distances. Most regions with

notable changes are far from their TSSs. (**D**) The distributions of the distances between ATAC-seq peaks (all peaks, CAAs, and CIAs) and the TSS. (**E**) Annotations of CAAs and CIAs presenting the genomic features for the differentially accessible regions. (**F**) The relative levels of H3K4me3 in PBMCs for randomly selected CAAs and CIAs as measured using ChIP-qPCR. The enrichment of H3K4me1 is normalized to 10% input, and IgG is used as a negative control. Error bars represent the means ± S.E.M for three independent experiments. $^{**}p < 0.01$, $^{***}p < 0.001$, as determined using one-way ANOVA with Dunnett's multiple comparisons test.

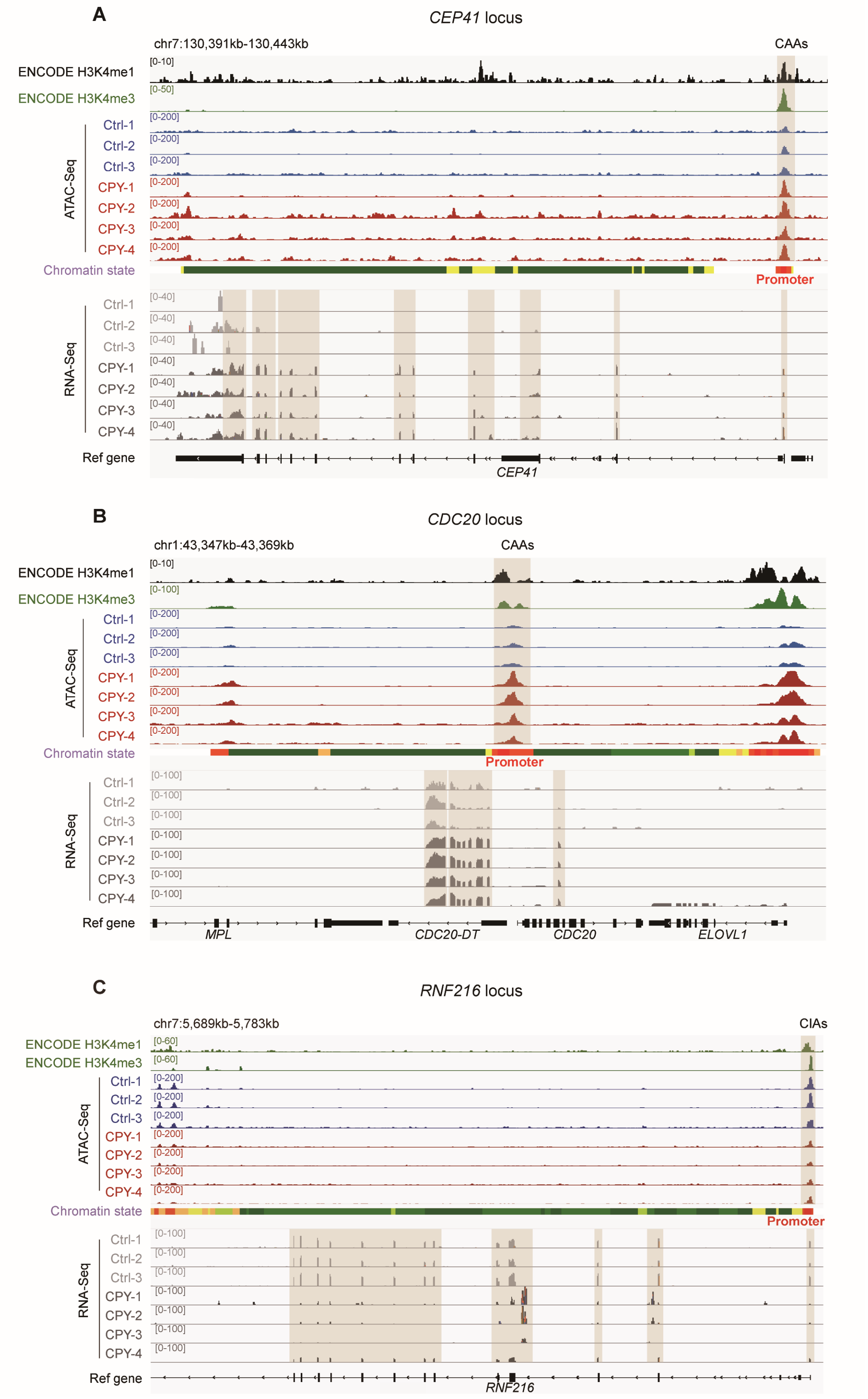
A
CEP41 locus
chr7:130,391kb-130,443kb
CAAs
ENCODE H3K4me1
ENCODE H3K4me3
ATAC-Seq
Ctrl-1
Ctrl-2
Ctrl-3
CPY-1
CPY-2
CPY-3
CPY-4
Chromatin state
Promoter
RNA-Seq
Ref gene
CEP41
B
CDC20 locus
chr1:43,347kb-43,369kb
CAAs
Promoter
MPL
CDC20-DT
CDC20
ELOVL1
C
RNF216 locus
chr7:5,689kb-5,783kb
CIAs
RNF216

Supplementary Figure S5. CAAs are linked to expression of distinct cilia genes. (**A-C**) Integrated Genomic Viewer (IGV) snapshot displaying the H3K4me1 and H3K4me3 signals of PBMCs from ENCODE, and ATAC-seq, RNA-seq signals of PBMCs in (**A**) *CEP41,* (**B**) *CDC20*, and (**C**) *RNF216* gene loci. Vertical gray boxes indicate promoter region ATAC-seq signals and corresponding expression of cilia genes.

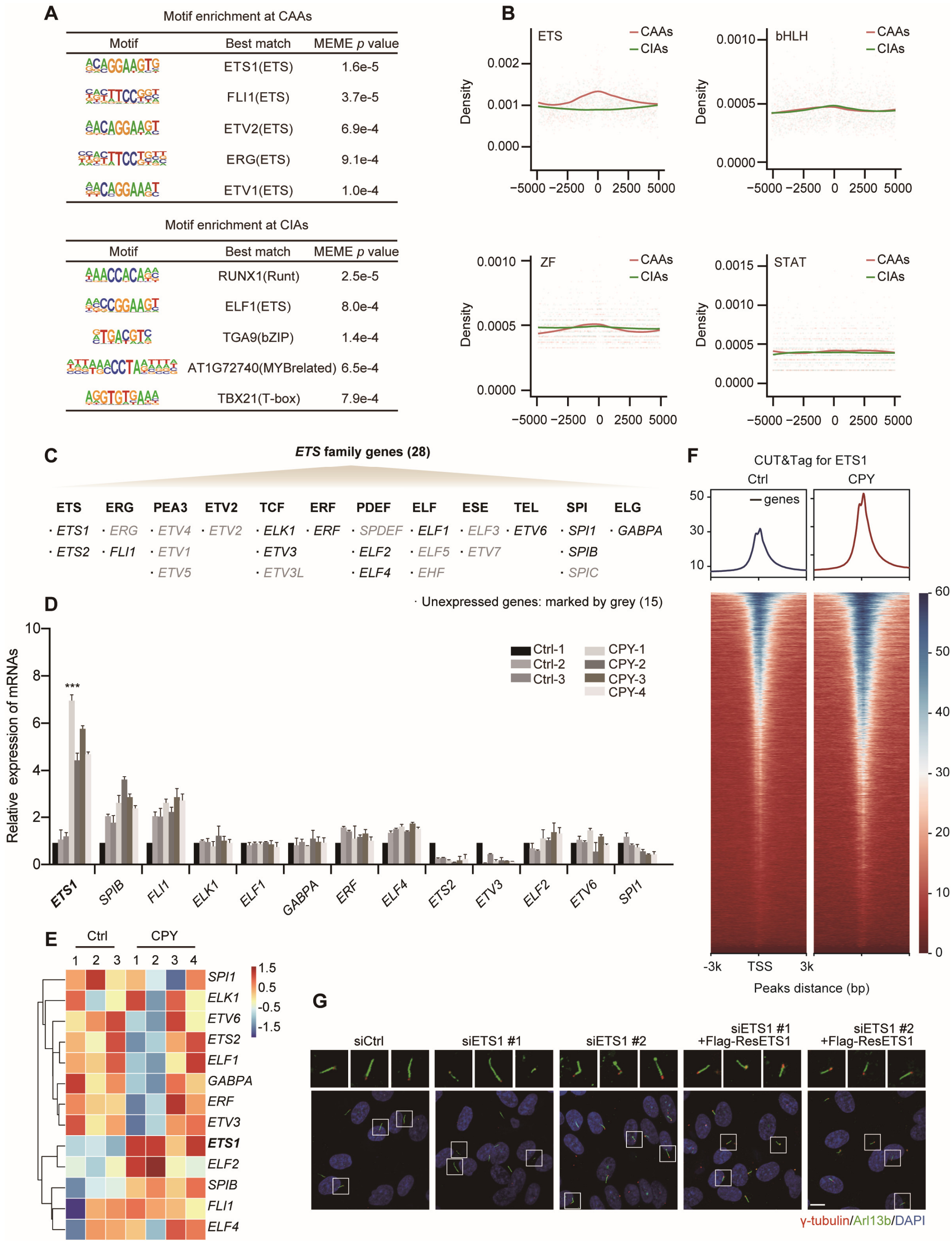

Supplementary Figure S6. ETS1 binds to CAAs to accelerate transcription of cilia genes. (**A**) Top-ranked enriched motifs from MEME motif analysis of CAAs and CIAs. The *p* value was calculated using the hypergeometric test (one-tailed). (**B**) Position-weight matrix showing the observed densities in four motif families containing ETS, bHLH, ZF, and STAT sites. (**C**) Summary schematic depicting the subgroup and member belonging to ETS motif family; The genes marked by gray presents the no-expression or low-expression genes of RNA-seq in PBMCs. (D) The relative expression of ETS family genes in Ctrl and CPY was measured by RT-qPCR. Error bars represent the means ± S.E.M for three independent experiments. $*p < 0.05$ as determined using Student's *t*-test. (**E**) Heatmap showing the expression of ETS family genes of RNA-seq in Ctrl and CPY. (**F**) Heatmaps and enrichment plots showing normalized read densities of ETS1 CUT&Tag signals of Ctrl and CPY performed in PBMCs. Tracks are centered at the TSS, extending ± 3 kb. **(G)** Immunostaining of γ-tubulin (red) and Arl13b (green) in control, siETS1-treated and Flag-ResETS1-rescued hTERT RPE-1 cells. DNA was stained with DAPI (blue). Scale bars: 5 μm.

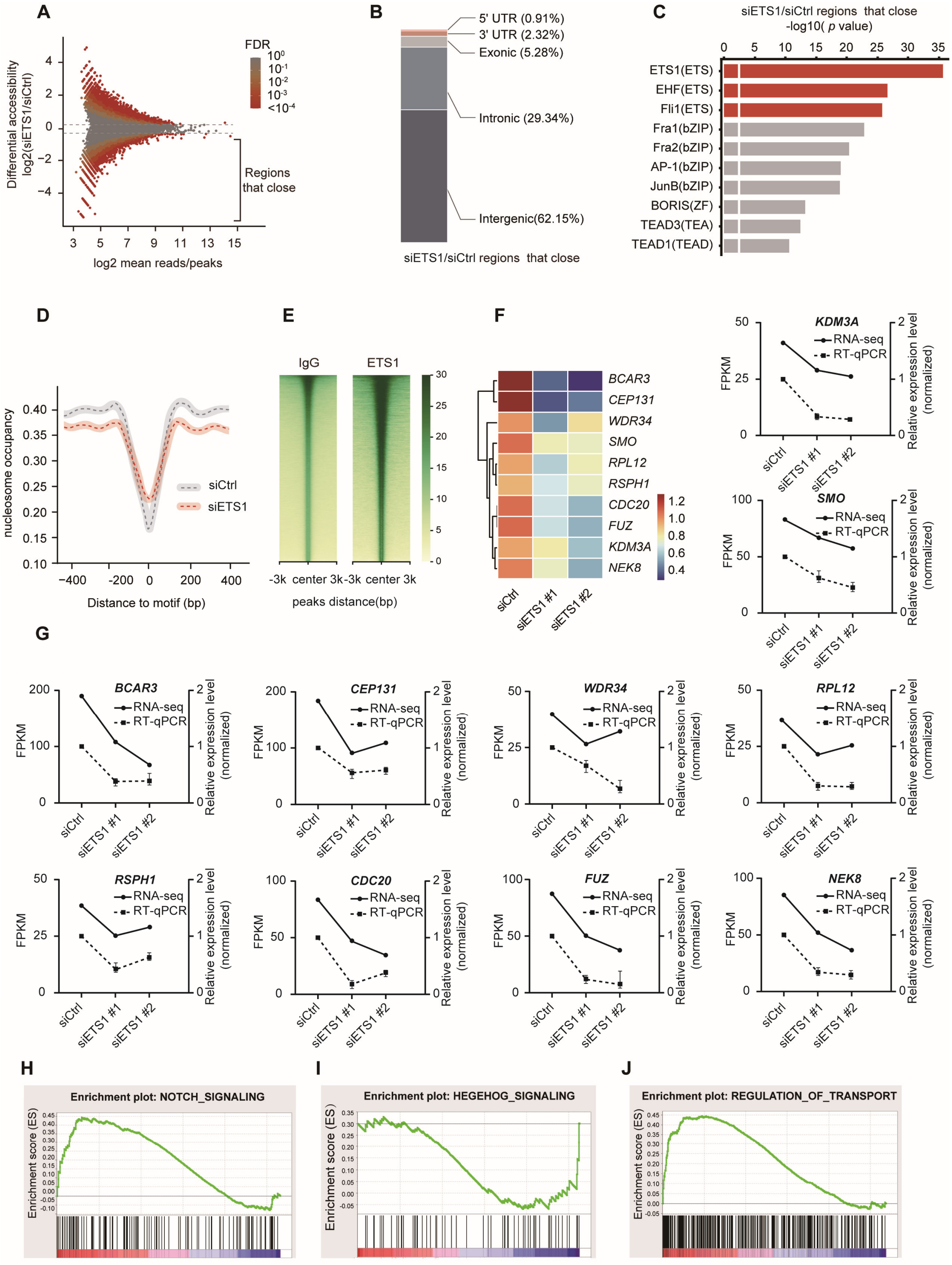

Supplementary Figure S7. Deletion of ETS1 reduces collapse of CAAs. (**A**) Differential accessibility of siETS1 versus siCtrl (log2 fold change in reads per accessible region) plotted against the mean reads per region. FDR is false discovery rate that ∣log2FC∣>1. (**B**) Annotations of genomic distribution of the peaks that close after ETS1 knockdown. (**C**) Gene Ontology enrichments of the regions that close in

siETS1 cells using Genomic Regions Enrichment of Annotations Tool (GREAT) analysis. Bar length represents the enriched *p* value for biological processes. (**D**) Nucleosome occupancy around ETS1 sites in siETS1 and siCtrl cells. Higher motif scores indicate increased likelihood of ETS1 binding. (**E**) Heatmaps showing normalized read densities of ETS1 CUT&Tag signals performed in hTERT RPE-1 cells. Tracks are centered at the peaks and extend ± 3 kb. (**F**) Heatmap displaying transcriptome change in expression of representative cilia genes after loss of ETS1. (**G**) The expression of *BCAR3*, *CEP131*, and eight other genes in hTERT RPE-1 cells after ETS1 knockdown. The dashed line indicates RT-qPCR results, and the solid line indicates RNA-seq data. Error bars represent the means ± S.E.M. for three independent experiments. (**H-J**) GSEA showing the enrichment of Hedgehog, Notch and regulation of transport in ETS1-knockdown cells.

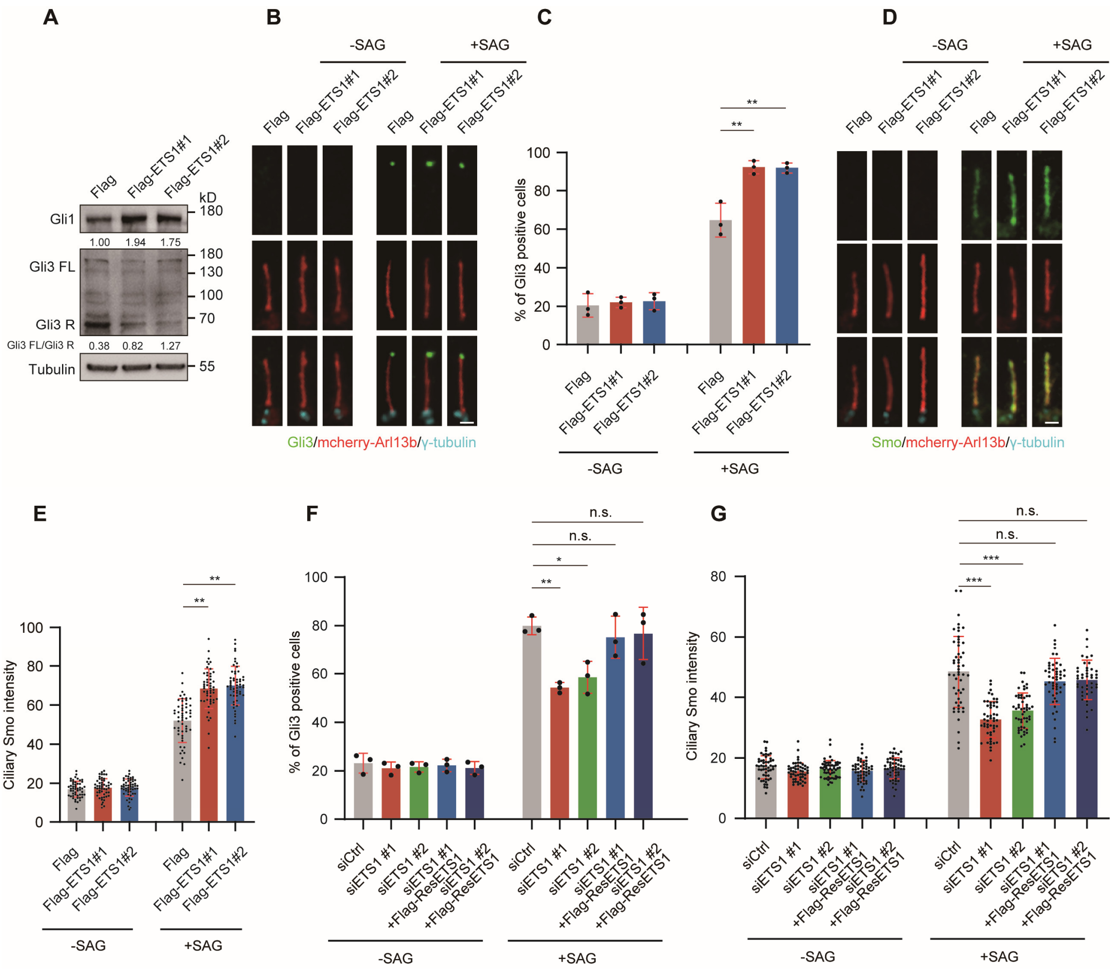

Supplementary Figure S8. ETS1 positively regulates hedgehog signaling transduction. (**A**) Immunoblots of Gli1 and Gli3 in control, ETS1 overexpressed hTERT RPE-1 cells. The intensity of Gli1 and the ratio of Gli3 FL/Gli3 R was quantified. Tubulin was used as a loading control. (**B**) Immunostaining of Gli3 (green) and γ-tubulin (cyan) in control and ETS1 overexpressed hTERT RPE-1 cells that transfected with mcherry-Arl13b with or without SAG. Scale bars: 1 μm. (**C**) Quantification of the percentage of Gli3 positive cells in control and ETS1-overexpressed hTERT RPE-1 cells with or without SAG activation from (B). (**D**) Immunostaining of Smo (green) and γ-tubulin (cyan) in control and ETS1 overexpressed hTERT RPE-1 cells that transfected with mcherry-Arl13b with or without SAG. Scale bars: 1 μm. (**E**) Quantification of ciliary Smo intensity (E) in control and ETS1-overexpressed hTERT RPE-1 cells with or without SAG activation from (D). (**F, G**) Quantification of the percentage of Gli3 positive cells (F) and ciliary Smo intensity (G) in control, siETS1-treated and Flag-ResETS1-rescued hTERT RPE-1 cells with or without SAG activation. For **C, E, F**, and **G**, error bars represent the means ± S.E.M for three independent experiments. n.s., not significant, **p* < 0.05, ***p* < 0.01, ****p* < 0.001, as determined using one-way ANOVA analysis.

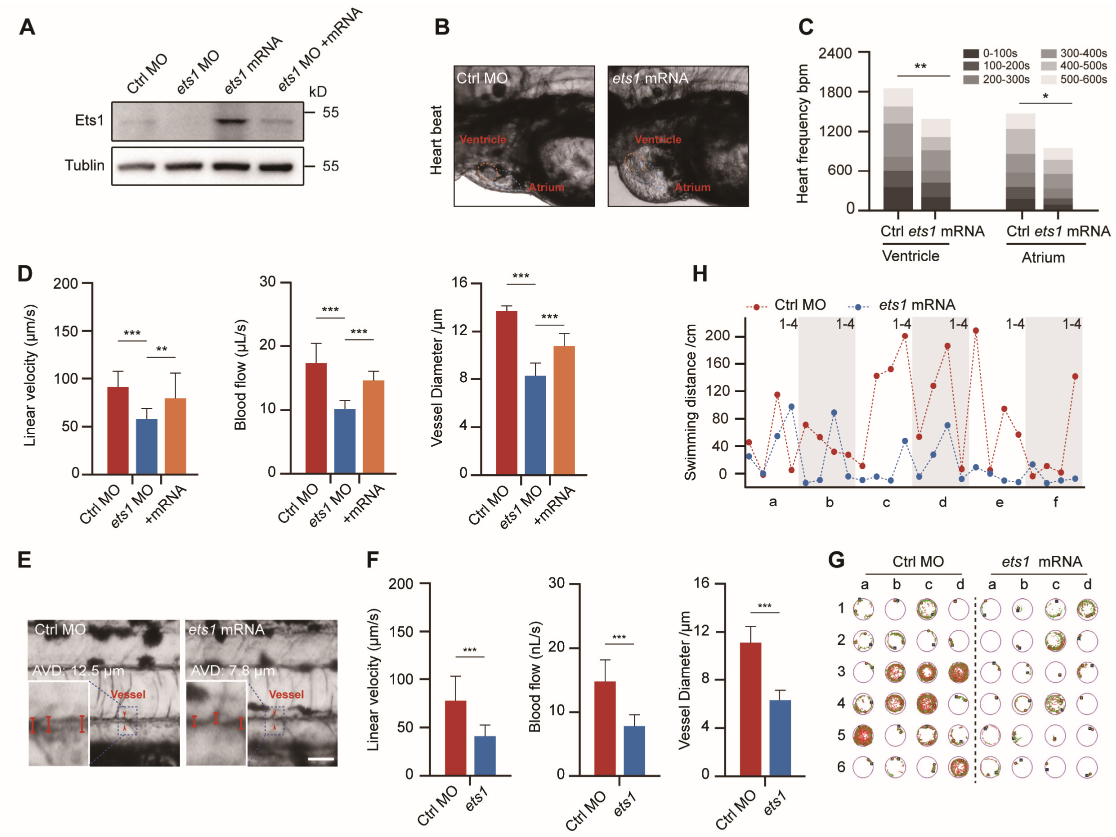

Supplementary Figure S9. Forced Ets1 expression leads to EVC ciliopathy phenotypes in zebrafish larvae. (**A**) Ets1 protein level was decreased obviously after ets1 morpholino injection. Injection of in vitro synthesized ets1 mRNA up-regulated Ets1 protein level. (**B, C**) The heart rate, expressed in beats per minute (bpm), in the ventricle and atrium over various time intervals for a total time of 10 min. The ventricle and atrium in control and forced *ets1* expression larvae are labeled by dash circles. *$p < 0.05$, **$p < 0.01$, as determined using Student's *t*-test. (**D**) The linear velocity, blood flow, and vessel diameter were measured using Zebra BloodFlow software. Error bars represent means ± S.E.M obtained from independent data captured in movie during a total time of 5 minutes. **$p < 0.01$, ***$p < 0.001$, as determined Student's *t*-test. (**E**) The representative blood flow dynamics map of control and forced *ets1* expression larvae at 5 dpf. The linear flow meter process was captured in a movie to show the change of blood flow in real time. AVD: Average vessel diameter. Scale bar, 100 μm. (**F**) The linear velocity, blood flow, and vessel diameter were measured using Zebra BloodFlow software. Error bars represent means ± S.E.M obtained from independent data captured in movie during a total time of 5 minutes. ***$p < 0.001$, as determined using Student's *t*-test. (**G, H**) Representative results of locomotion trajectories and swimming distance analysis in control and forced *ets1* expression at 5 dpf during a total time of 20 minutes. The green line represents the trajectory at moderate velocity, and the red line represents the trajectory at high velocity.

A

Rank	Motif	Name	-log$_{10}$(p value)	% of Targets with Motif
1	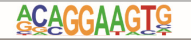	ETS1(ETS)/Jurkat-ETS1-ChIP-Seq(GSE17954)/Homer	28.66	55.52%
2	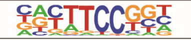	EWS:ERG-fusion(ETS)/CADO_ES1-EWS:ERG-ChIP-Seq(SRA014231)/Homer	28.03	63.37%
3	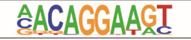	ERG(ETS)/VCaP-ERG-ChIP-Seq(GSE14097)/Homer	25.45	49.49%
4	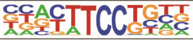	Etv2(ETS)/ES-ER71-ChIP-Seq(GSE59402)/Homer(0.967)	22.70	36.17%
5	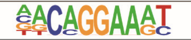	EWS:FLI1-fusion(ETS)/SK_N_MC-EWS:FLI1-ChIP-Seq(SRA014231)/Homer	20.72	55.72%
6	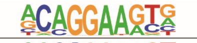	Ets1-distal(ETS)/CD4+-PolII-ChIP-Seq(Barski_et_al.)/Homer	17.02	42.46%
7	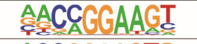	ETV1(ETS)/GIST48-ETV1-ChIP-Seq(GSE22441)/Homer	14.98	45.21%
8	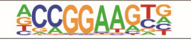	ETV4(ETS)/HepG2-ETV4-ChIP-Seq(ENCODE)/Homer	12.33	30.81%
9	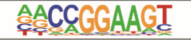	GABPA(ETS)/Jurkat-GABPa-ChIP-Seq(GSE17954)/Homer	11.34	17.94%
10	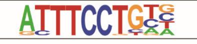	Fli1(ETS)/CD8-FLI-ChIP-Seq(GSE20898)/Homer	10.33	18.78%
......				
33	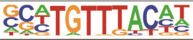	FOXK2(Forkhead)/U2OS-FOXK2-ChIP-Seq(E-MTAB-2204)/Homer	3.31	9.48%
52	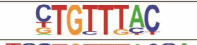	Foxo1(Forkhead)/RAW-Foxo1-ChIP-Seq(Fan_et_al.)/Homer	2.54	1.03%
61	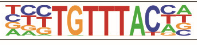	FOXP1(Forkhead)/H9-FOXP1-ChIP-Seq(GSE31006)/Homer	2.06	5.92%

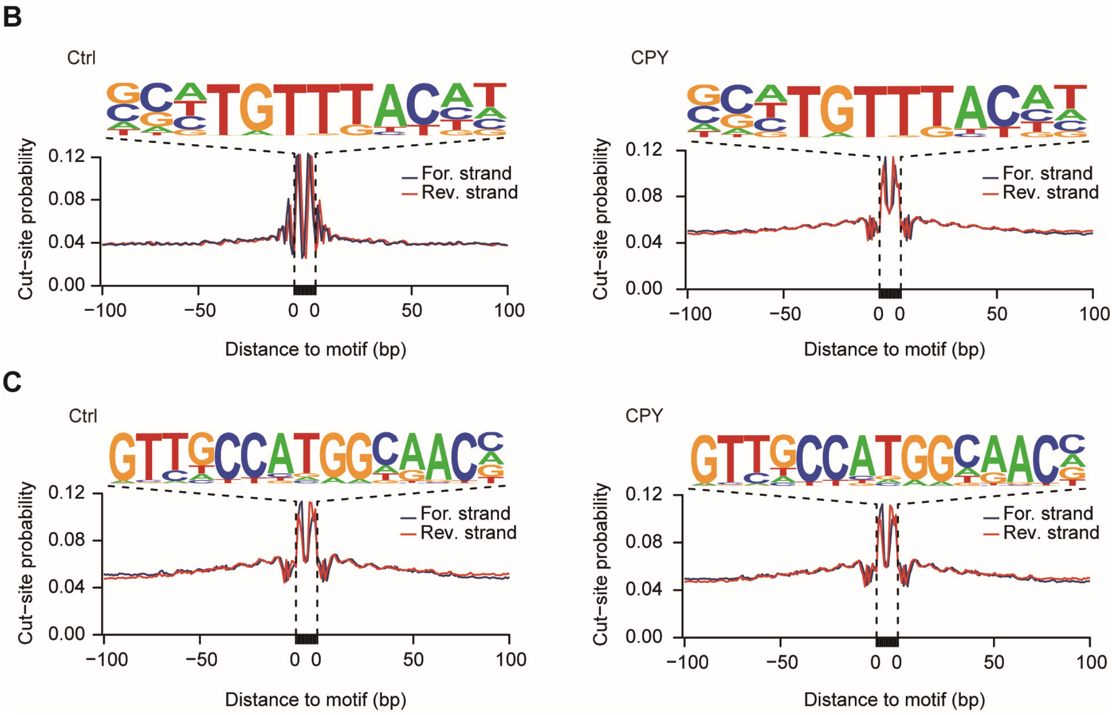

Supplementary Figure S10. Recruitment of Forkhead and RFX motifs on CAAs regions. (**A**) Top-ranked enriched motifs of CAAs using Homer2 algorithms. The p-value was calculated using the hypergeometric test (one-tailed). (**B**, **C**) ATAC-seq footprint at the Forkhead (**B**) and RFX (**C**) motif site in Ctrl and CPY. Insertions per site are normalized to have the same average number of insertions 200–500 bp away from the motif.

REFERENCES

1. Kinkley, S., Helmuth, J., Polansky, J.K., Dunkel, I., Gasparoni, G., Fröhler, S., Chen, W., Walter, J., Hamann, A. and Chung, H.R. (2016) reChIP-seq reveals widespread bivalency of H3K4me3 and H3K27me3 in CD4(+) memory T cells. Nat Commun, 7, 12514.
2. Xiao, F.H., Yu, Q., Deng, Z.L., Yang, K., Ye, Y., Ge, M.X., Yan, D., Wang, H.T., Chen, X.Q., Yang, L.Q. et al. (2022) ETS1 acts as a regulator of human healthy aging via decreasing ribosomal activity. Sci Adv, 8, eabf2017.
